# Comparative genomic and functional analyses of *Paenibacillus peoriae* ZBSF16 with biocontrol potential against grapevine diseases, provide insights into its genes related to plant growth-promoting and biocontrol mechanisms

**DOI:** 10.3389/fmicb.2022.975344

**Published:** 2022-09-08

**Authors:** Lifang Yuan, Hang Jiang, Xilong Jiang, Tinggang Li, Ping Lu, Xiangtian Yin, Yanfeng Wei

**Affiliations:** ^1^Shandong Academy of Grape, Shandong Academy of Agricultural Sciences, Jinan, Shandong, China; ^2^Institute of Plant Protection, Shandong Academy of Agricultural Sciences, Jinan, Shandong, China; ^3^College of Advanced Agricultural Sciences, Zhejiang A&F University, Hangzhou, Zhejiang, China

**Keywords:** *Paenibacillus peoriae*, comparative genome analysis, plant growth-promoting, biocontrol, antimicrobial substances

## Abstract

*Paenibacillus peoriae* is a plant growth-promoting rhizobacteria (PGPR) widely distributed in various environments. *P. peoriae* ZBFS16 was isolated from the wheat rhizosphere and significantly suppressed grape white rot disease caused by *Coniella vitis*. Here, we present the complete genome sequence of *P. peoriae* ZBFS16, which consists of a 5.83 Mb circular chromosome with an average G + C content of 45.62%. Phylogenetic analyses showed that ZBFS16 belongs to the genus *P. peoriae* and was similar to *P. peoriae* ZF390, *P. peoriae* HS311 and *P. peoriae* HJ-2. Comparative analysis with three closely related sequenced strains of *P. peoriae* identified the conservation of genes involved in indole-3-acetic acid production, phosphate solubilization, nitrogen fixation, biofilm formation, flagella and chemotaxis, quorum-sensing systems, two-component systems, antimicrobial substances and resistance inducers. Meanwhile, *in vitro* experiments were also performed to confirm these functions. In addition, the strong colonization ability of *P. peoriae* ZBFS16 was observed in soil, which provides it with great potential for use in agriculture as a PGPR. This study will be helpful for further studies of *P. peoriae* on the mechanisms of plant growth promotion and biocontrol.

## Introduction

*Paenibacillus peoriae* (previously *Bacillus peoriae*) is a Gram-positive, facultatively anaerobic, rod-shaped bacterium with flagella and belongs to the genus *Paenibacillus* and the family *Paenibacillaceae*. Species in the genus *Paenibacillus* are either Gram-positive or variable, facultatively anaerobic or strictly aerobic, produce ellipsoidal endospores, and are nonpigmented, rod-shaped and motile (Ash et al., 1993; Siddiqi et al., 2015). Currently, the genus *Paenibacillus* contains 240 species, including the plant-beneficial species of *P. polymyxa* (Zhang et al., 2018; Timmusk et al., 2019), *P. ehimensis* (Naing et al., 2015), *P. alvei* (Emmanouil et al., 2016), *P. macerans* (Liang et al., 2014), *P. lentimorbus* (DasGupta et al., 2006) and *P. peoriae* (Von der Weid et al., 2003; Jiang et al., 2022). Previously, *P. peoriae* was reported to act as a plant growth-promoting rhizobacteria (PGPR), which can produce biofilms, stably colonize the rhizosphere of plants and compete with other microbiota (Von der Weid et al., 2003; Vejan et al., 2016; Jiang et al., 2022). Meanwhile, *P. peoriae* has the ability to act as a biological control agent against many plant pathogens, including *Fusarium* spp., *Diplodia macrospora*, *D. maydis*, *Verticillium dahlia*, *Rhizoctonia solani*, *Colletotrichum gloeosporioides*, and *C. graminicola* (Von der Weid et al., 2003; Yadav D. et al., 2021; Jiang et al., 2022), and even the antimicrobial peptide purified from *P. peoriae* could protect against *Staphylococcus aureus*, *Escherichia coli*, and *Candida albicans* (Ngashangva et al., 2021).

PGPR has been considered environmentally friendly alternatives to fertilizers or agrochemicals for improving crop yield and quality (Vejan et al., 2016; Hashem et al., 2019). Many microorganisms, such as *Bacillus*, *Pseudomonas*, *Burkholderia*, *Caulobacter*, and *Paenibacillus* spp., are PGPRs, and some have or will be successfully applied in practical applications (Ahemad, 2015; Garcia-Seco et al., 2015; Hashem et al., 2019). Production of indole-3-acetic acid (IAA), the capability of fixation of nitrogen, dissolution of phosphorus, secretion of ferriphagin and plant hormones, and antibiotic biosynthesis are important mechanisms of PGPR (Li et al., 2020; Yin et al., 2022). IAA is an important phytohormone that controls cell enlargement and tissue differentiation of plants. Nitrogen (N) and phosphorus (P) are important nutrients for plant growth and productivity. PGPRs are called diazotrophs because of their ability to fix N_2_ in nonleguminous plants and form a nonobligate interaction with host plants (Ahemad, 2015). Additionally, by providing P to plants, PGPRs solubilize inorganic P in soil to low molecular weight organic acids (Zaidi et al., 2009; Yuan et al., 2020). Siderophores can form stable complexes with Fe and other heavy metals (Al, Cd, Cu, Ga, In, Pb and Zn), and most plant growth promotion occurs *via* siderophore-mediated Fe uptake (Rajkumar et al., 2010). *P. polymyxa*., which is closest to *P. peoriae*, was identified as having key genes or gene clusters related to IAA, phosphate solubilization and nitrogen fixation for plant growth promotion (Li et al., 2020; Zhou et al., 2020).

The predominant genera of PGPRs are *Pseudomonas* and *Bacillus*, which have the feature of biocontrol, as well as most species in *Paenibacillus* (Naing et al., 2015; Grady et al., 2016; Hashem et al., 2019)*. Paenibacillus* helps to control phytopathogens (bacteria, fungi, nematodes and viruses) by triggering induced systemic resistance (ISR) by producing secondary metabolites (Grady et al., 2016). Antimicrobial substances produced by *Paenibacillus*, including peptides, enzymes, and volatile organic compounds, could be used to control soil-borne fungal pathogens and food-borne bacteria (Zhai et al., 2021). Paenicidin A and penisin are antimicrobial peptides produced by *P. polymyxa* NRRL B-30509 and *Paenibacillus* sp. strain A3, respectively (Baindara et al., 2015; Van Belkum et al., 2015). Paenibacillin exhibits excellent tolerance to pH and heat, with activity against a broad range of fungi and bacteria (Abriouel et al., 2011; Li Y. et al., 2019; Li L. et al., 2019). Nonribosomal peptide synthetases are large multimodular biocatalysts that utilize complex regiospecific and stereospecific reactions to assemble structurally and functionally diverse peptides that have important medicinal applications (Strieker et al., 2010).

The role of *P. peoriae* in plant growth promotion and biological control remained unexplored until very recently, and few reports revealed the mechanisms regarding the plant growth promotion and biological control of *P. peoriae. P. peoriae* ZBSF16 exhibit significant broad inhibitory spectra against various pathogenic fungi and bacteria on grape and possess perfect characteristics and potential for the biocontrol of grape diseases. In this study, we demonstrated the sequence and annotation of *P. peoriae* strain ZBSF16 and compared its genome with the three major representative *P. peoriae* strains (*P. peoriae* ZF390, *P. peoriae* HS311 and *P. peoriae* HJ-2) that are beneficial to plant growth. Our aim was to provide important insights into the functions of the biocontrol strains and analyze the mechanisms of plant growth promotion and biological control at the gene level, which will benefit improved application of *P. peoriae* to plants in the field.

## Materials and methods

### Bacterial strains, culture conditions, antagonistic assays and genomic DNA extraction

*P. peoriae* ZBSF16 was isolated from the wheat rhizosphere in Shandong Province, China on May 7, 2020 and was deposited as a reference strain (strain no. 24769) in the China General Microbiological Culture Collection Center. Strain ZBSF16 was cultivated in LB (Luria broth) medium at 28°C with shaking at 180 rpm for 24 h. The growth curve and the dynamic change in pH were measured every 4 h by spectrophotometer (Persee, TU-1900) and pH meter (Sartorius, PB-10) and the biochemical tests were performed as described by Yin et al. (2022). The morphology of the strains was observed scanning electron microscope (TESCAN VEGA3 SBU). Strain ZBSF16 was evaluated for its antagonistic activities to *Coniella vitis*, *Gloeosporium fructigrum, Pestalotiopsis clavispora*, *Alternaria viticola*, *Diaporthe eres*, *F. oxysporum*, *Botrytis cinerea*, *Botryosphaeria dothidea*, *Aspergillus niger*, *F. graminearum*, *F. pseudograminearum* and *Allorhizobium vitis* by plate bioassays inoculated with 2 μl of bacterial suspension (Li Y. et al., 2019). The inoculation concentration of strain ZBSF16 was determined by the optical density at 600 nm (OD_600_ = 0.8). Genomic DNA was extracted from cultured ZBSF16 cells (OD_600_ = 0.8) using a QIAamp® DNA Mini Kit (Qiagen, Valencia, CA, United States) according to the manufacturer’s instructions.

### Whole-genome sequencing and assembly

The genomic DNA of *P. peoriae* ZBSF16 was sequenced at Biomarker Technologies with the Pacific Biosciences (PacBio) RSII Single Molecule Real Time (SMRT) sequencing platform (Li Y. et al., 2019). For genome assembly, the filtered subreads were assembled by Canu v1.5 software, and then, circlator v1.5.5 was used to cyclize the assembled genome. A 10-kb insert size template library was prepared according to the PacBio Sequel gDNA protocol and sequenced using the PacBio Sequel instrument. Circular genome views of the alignments were generated by CGView (Yuan et al., 2020).

### Gene prediction and functional annotation

Genes and components of the genome were predicted by using Prodigal v2.6.3, and functional annotation was performed by comparisons against multiple databases, including NR (nonredundant) protein databases, SwissProt and the enhanced COG database, KEGG database, TrEMBL, and the Eggnog database. Transfer RNA (tRNA) genes were predicted with tRNAscan-SE v2.0, and ribosome RNA (rRNA) genes were predicted with Infernal v1.1.3. antiSMASH v5.0.0 was used to predict secondary metabolic gene clusters, and CRT v1.2 was used for CRISPR identification. Furthermore, pathogenicity and drug resistance can be researched by BLAST against the CAZy, TCDB, CARD, PHI, and VFDB databases.

### Phylogenetic tree construction

The evolutionary position of *P. peoriae* ZBSF16 was determined by 16S rDNA gene sequence analysis, multilocus sequence analysis (MLSA) and PhyloPhlAn method (Segata et al., 2013; Asnicar et al., 2020; Yin et al., 2022). 22 strains belonging to *Paenibacillus* were selected for constructing phylogenetic trees to investigate the evolution of strain ZBSF16 (Supplementary Table 1). Five housekeeping genes (16S rRNA, *gyrB*, *rpoD*, *rho*, and *pgk*) were selected for MLSA, sequence alignments of ZBSF16 with other *Paenibacillus* strains were carried out using the maximum likelihood clustering method, which was performed in MEGA6 with a bootstrapping test of 1,000 replications to generate phylogenetic trees.

### Comparative genomics analysis and mining for genes related to plant-beneficial traits

For the comparative genomic analysis, the genome sequences of *P. peoriae* ZBSF16 were compared to *P. peoriae* ZF390, *P. peoriae* HS311 and *P. peoriae* HJ-2 by MAUVE comparison software (Darling et al., 2004). Additionally, a circular chromosomal map of all the genomes used in the pan-genome analysis was prepared by using BLAST Ring Image Generator (BRIG) v 0.95, taking strain ZBSF16 as a reference genome (Alikhan et al., 2011; Mukhia et al., 2022). Furthermore, average nucleotide identity (ANI) was conducted by using the orthologous average nucleotide identity (OrthoANI) tool, and *in silico* DNA–DNA hybridization (DDH) was calculated by using the Genome-to-Genome Distance Calculator (GGDC) (Goris et al., 2007). Functional genes involved in plant growth promotion, such as genes responsible for IAA production, phosphate solubilization, nitrogen fixation, biofilm formation and synthesis resistance inducers, were searched in the NCBI databases as described by Kumar et al. (2019). The blast search was performed against the locally constructed database of the publically available genomes of *P. peoriae*, with the genome of *P. peoriae* ZBSF16 as a query. The identities of different functional genes at the amino acid level were compared among the strains by using BLAST, with an E-value cut off of 1e-15 was used for the BLAST search (Kumar et al., 2019). Secondary metabolite gene clusters were predicted by antiSMASH 4.0.2 (Jiang et al., 2022).

### Measurement of IAA production, phosphate solubilization, siderophores and ammonia production

To determine the production of IAA, strain ZBSF16 was cultured in DF (peptone, 5.0 g; yeast extract, 1.5 g; beef extract, 1.5 g/l; NaCl, 5.0 g/l; tryptophan, 0.5 g/l) salt minimal medium, with a concentration of L-tryptophan of 1.02 g/l. After incubation for 24 h at 28°C, the IAA concentration was estimated as the method described by Yuan et al., (2020). The capability of strain ZBSF16 to solubilize phosphate was estimated *via* National Botanical Research Institute Phosphate (NBRIP) solid medium as described by Yin et al., (2022), and the clear zone around the colony was measured after 7 days at 28°C. A CAS agar plate was used for qualitative analysis of siderophores, and yellow circles that appeared around the colonies were measured after 7 days at 28°C. The capability of strain ZBSF16 to produce ammonia was detected by the method described by Przemienieck, and Nessler’s reagent was used to determine its ability to produce ammonium (Przemieniecki et al., 2019; Elhaissoufi et al., 2020).

### Analyses of antibiotic resistance and hemolysis

The characteristics of antibiotic resistance of strain ZBSF16 were tested on nine antibiotics, including ampicillin (200 μg/ml), kanamycin (50 μg/ml), rifampicin (50 μg/ml), vancomycin (50 μg/ml), streptomycin (10 μg/ml), spectinomycin (50 μg/ml), gentamycin (10 μg/ml), tetracycline (5 μg/ml), and chloramphenicol (20 μg/ml). The minimum inhibitory concentration (MIC) and minimum bactericidal concentration (MBC) of spectinomycin for strain ZBSF16 were determined as previously described. *P. peoriae* ZBSF16 was grown in LB broth at 28°C for 24 h, Wagstsuma Blood Agar Base (Hopebio, China) was used to determine hemolysis as described previously (Brillard et al., 2001; Yuan et al., 2020).

### Plant growth promotion, colonization and biocontrol assays

To determine the plant growth promotion capability of ZBSF16, ten *Vitis vinifera* seedlings (cv. Red globe) were treated with 50 ml of ZBSF16 culture (10^8^ CFU/ml) by irrigation every 15 days for 2 months. Another ten *V. vinifera* seedlings used as controls were treated with sterile water. All treated grape plants were placed in a greenhouse maintained at temperature 28°C and 90% relative humidity (RH). At 60 days after inoculation, the root length, shoot length, fresh weight, and dry weight of the seedlings were measured. Meanwhile, the infection rate and disease index of grape white rot on *Vitis vinifera* seedlings (cv. Red globe) were calculated after inoculating *C. vitis* conidial suspension (10^6^ conidial/ml) two month later at 28°C and 70–80% RH (Chethana et al., 2017; Ji et al., 2021).

To observe the population dynamics of the ZBSF16 strain in the rhizosphere soil, *Vitis vinifera* seedlings (cv. Red globe) were transplanted into nursery pots containing sterile soil, and each seedling was irrigated with 50 ml of *P. peoriae* ZBSF16 bacterial suspension at a concentration of 10^8^ CFU ml^−1^. Rhizosphere soil was collected at different time points (0, 7, 14, 21, 28, 35, 42, 49 and 56 days after inoculation), and the number of ZBSF16 in the rhizosphere soil was determined by the plating counting method with LB medium containing spectinomycin and streptomycin.

Grape white rot caused by *Coniella vitis* was used as the pathosystem to determine the biocontrol potential of ZBSF16. Leaves and fruit of *V. vinifera* (cv. Red globe) were used to assess the preventive effect and control effect of strain ZBSF16 as described by Yin et al. (2022). Ten biological replicates were performed for each treatment, and the experiments were independently repeated three times. All the leaves and fruit were maintained at 28°C and 90% RH.

### Statistical analysis

All experimental data were analyzed by SPSS 22.0 software, and all the values are presented as the mean ± standard error of at least three replications. Significant differences (*p* < 0.05) were determined by one-way analysis (ANOVA) of variance and Duncan’s multiple range test (Yuan et al., 2020; Yin et al., 2022).

## Results

### Organism information and antagonistic characteristics

As a gram-positive, anaerobic, rod-shaped bacterium with a length of 3–5 μm and a diameter of 0.8–1.2 μm, ZBSF16 can utilize diverse carbon sources and belongs to the *Paenibacillus* genus (Supplementary Figures 1A,B; Supplementary Table 2). The growth curve showed that the strain was in the exponential growth phase between 4 and 20 h after inoculation, with the pH value increasing to 7.77 (Supplementary Figure 1C). Additionally, the strain grew best when the pH value was between 6 and 8 and could endure 2% NaCl (Supplementary Figures 1G,H).

**Figure 1:**
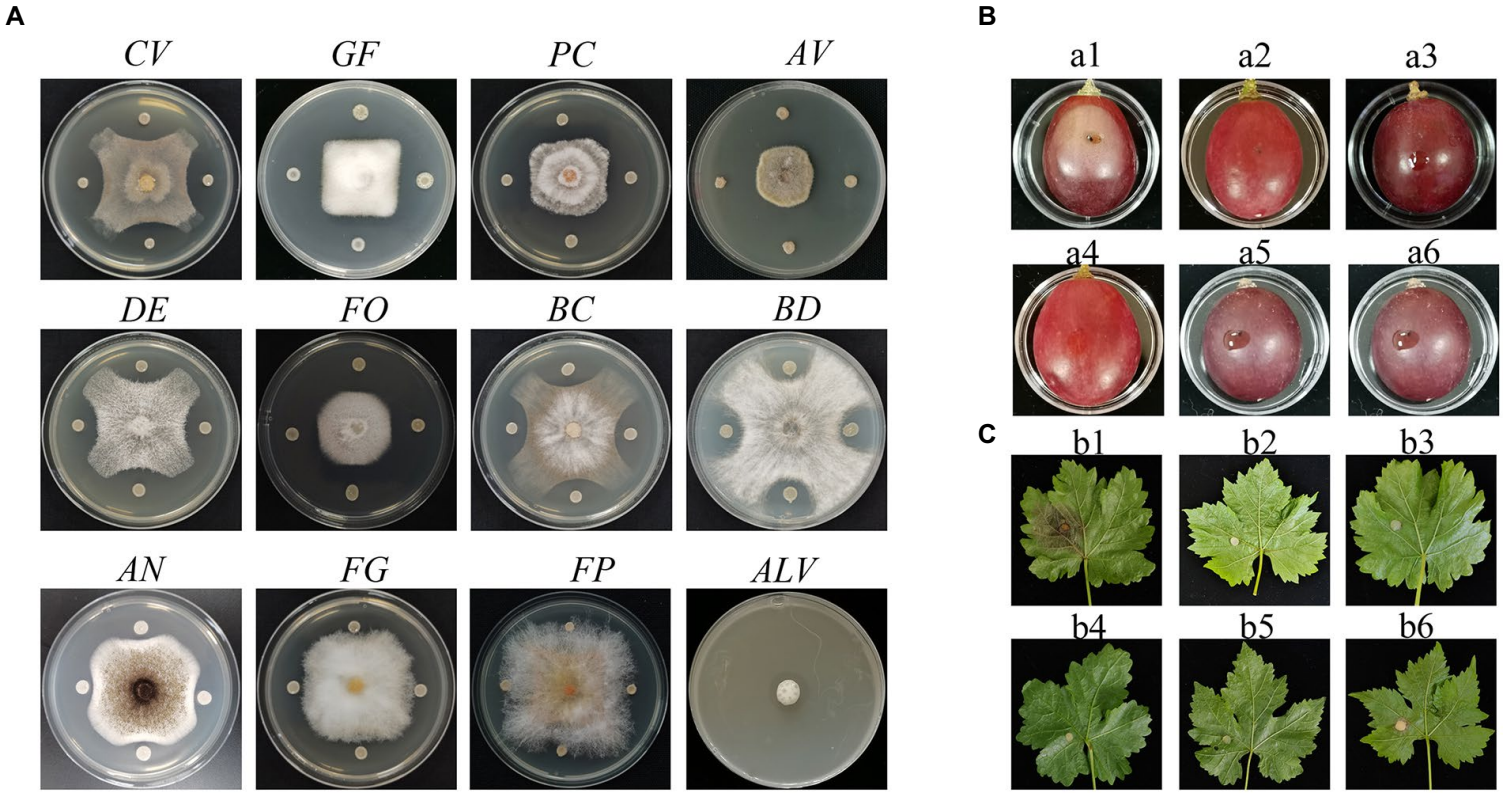
Antagonistic assay of *Paenibacillus peoriae* ZBSF16 against eleven pathogenic fungi and one pathogenic bacterium. **(A)** Antagonistic assay of *P. peoriae* ZBSF16. *Coniella vitis* (CV). *Gloeosporium fructigrum* (GF). *Pestalotiopsis clavispora* (Pc). *Alternaria viticola* (Av). *Diaporthe eres* (DE). *Fusarium oxysporum* (FO). *Botrytis cinerea* (BC)*. Botryosphaeria dothidea* (BD). *Aspergillus niger* (AN). *Fusarium graminearum* (FG). *Fusarium pseudograminearum* (FP). *Allorhizobium vitis* (ALV). **(B,C)** Biocontrol efficiency of *P. peoriae* ZBSF16 on grape white rot caused by *Coniella vitis*. (a1, b1) Inoculated with *C. vitis*; (a2, b2) LB broth; (a3, b3) sterile water; (a4, b4) culture of ZBSF16; (a5, b5) inoculated with *C. vitis* 24 h after inoculation with the culture of ZBSF16; (a6, b6) inoculated culture of ZBSF16 24 h after inoculation with *C. vitis*.

*P. peoria* ZBSF16 was isolated as a biocontrol agent for use against *Coniella vitis*, which exhibited the highest inhibitory rate of 64.44% (Supplementary Figure 2A). Antagonistic spectrum assays showed that strain ZBSF16 presented broad, strong antipathogenic activities against various fungi on grape, including *Gloeosporium fructigrum*, *Botrytis cinerea*, *Diaporthe eres*, *Alternaria viticola*, *F. oxysporum*, *Aspergillus niger*, *Pestalotiopsis clavispora*, and *Allorhizobium vitis* (Figure 1A). In addition, ZBSF16 is considered a biocontrol agent for its extracellular enzyme activity, and it can produce protease, cellulase and lipoidase, which is an important mechanism for inhibiting pathogens (Supplementary Figures 1D–F).

### Plant growth promotion, colonization and biocontrol assays

**Figure 2:**
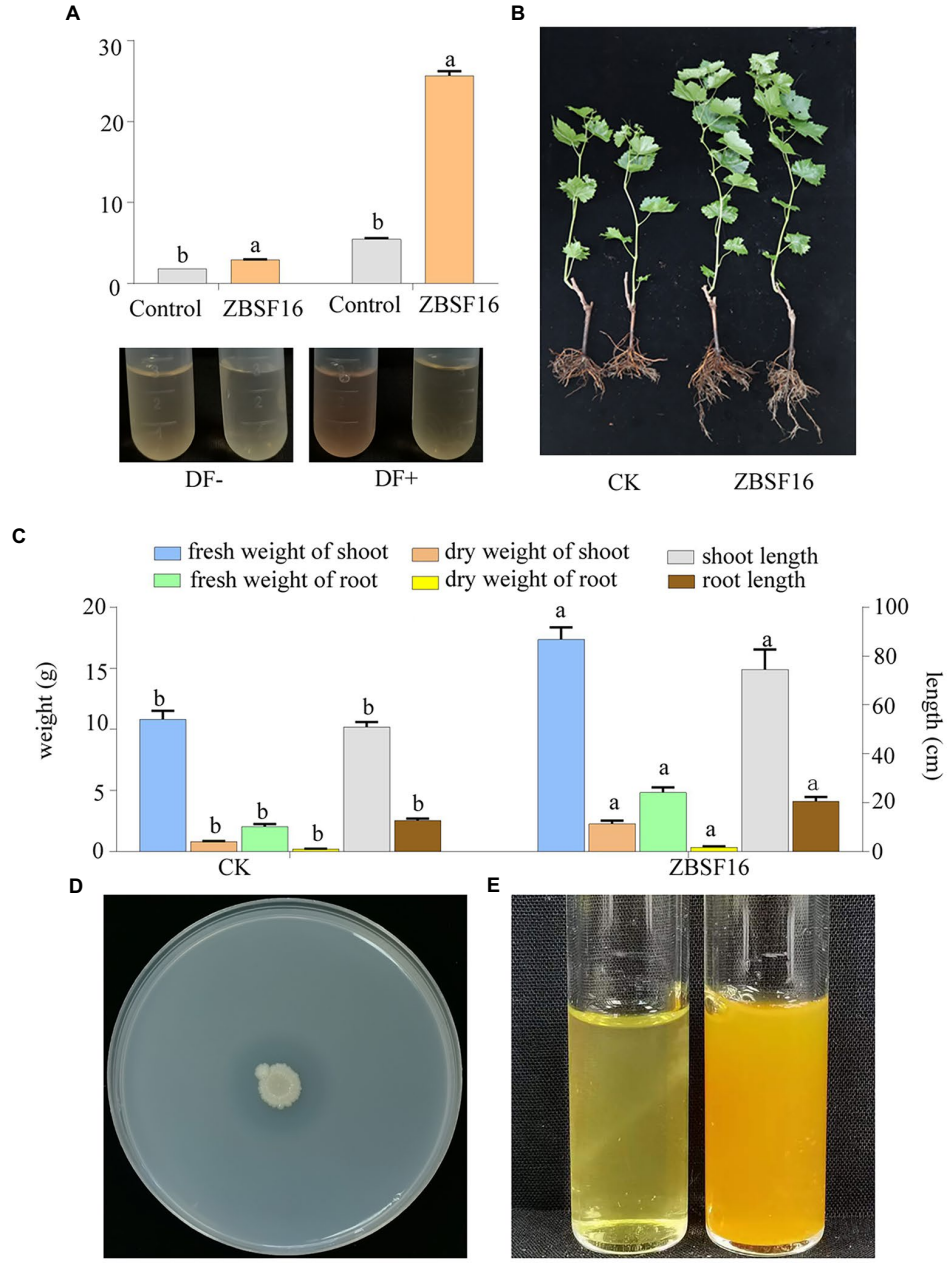
Determination of the plant growth-promoting properties of *Paenibacillus peoriae* ZBSF16. **(A)** IAA production of *P. peoriae* ZBSF16. DF−, DF medium without L-tryptophan; DF+, DF medium containing L-tryptophan. **(B,C)** The growth-promoting effect of *Paenibacillus peoriae* ZBSF16 on grape; **(D)** mineral phosphate solubilization of *P. peoriae* ZBSF16; **(E)** ammonia production of *P. peoriae* ZBSF16.

The ability of ZBSF16 to promote growth was verified by inoculating the rhizosphere of plants of *V. vinifera* (cv. Red globe) with the suspension in the greenhouse. *P. peoriae* ZBSF16 produced siderophores and was considered an excellent PGRP (Supplementary Figure 3E). The rate of growth promotion for the length (weight) of the aboveground parts and the root length (fresh weight, dry weight) were 46.56% (60.20, 183.75%) and 60.78% (137.25, 454.54%), respectively (Figure 2C). In addition, the bacterial counts of ZBSF16 on the root surface were maintained at 10^5^ CFU/g after 1 month of inoculation (Supplementary Figure 3F). Further study showed that the infection rate and disease index of grape white rot on *V. vinifera* caused by *C. vitis* were decreased 70% and 62.97, inoculating with strain ZBSF16 compared to the control plants (Supplementary Figures 2D,E).

Two treatments were performed to determine the preventive effect and control effect of strain ZBSF16. The results demonstrated that strain ZBSF16 displayed excellent biocontrol traits for grape white rot disease (Figures 1B,C), with the preventive effects for detached leaf and detached fruit being 90.59 and 94.52%, respectively. The control effects for detached leaves and detached fruit were 94.52 and 84.70%, respectively (Supplementary Figures 2B,C).

### Analyses of antibiotic resistance and hemolysis

The strain ZBSF16 exhibited resistance to ampicillin, chloramphenicol, tetracycline, gentamycin, rifampicin, kanamycin and vancomycin but not to streptomycin or spectinomycin. In addition, strain ZBSF16 showed an MIC of spectinomycin of 216 μg/ml and an MBC of 1,024 μg/ml (Supplementary Figures 3A–C). Meanwhile, the strain was unable to produce hemolysin activity on plates according to the blood agar hemolysis assay (Supplementary Figure 3D).

### General genomic features of *Paenibacillus peoriae* ZBSF16

**Figure 3:**
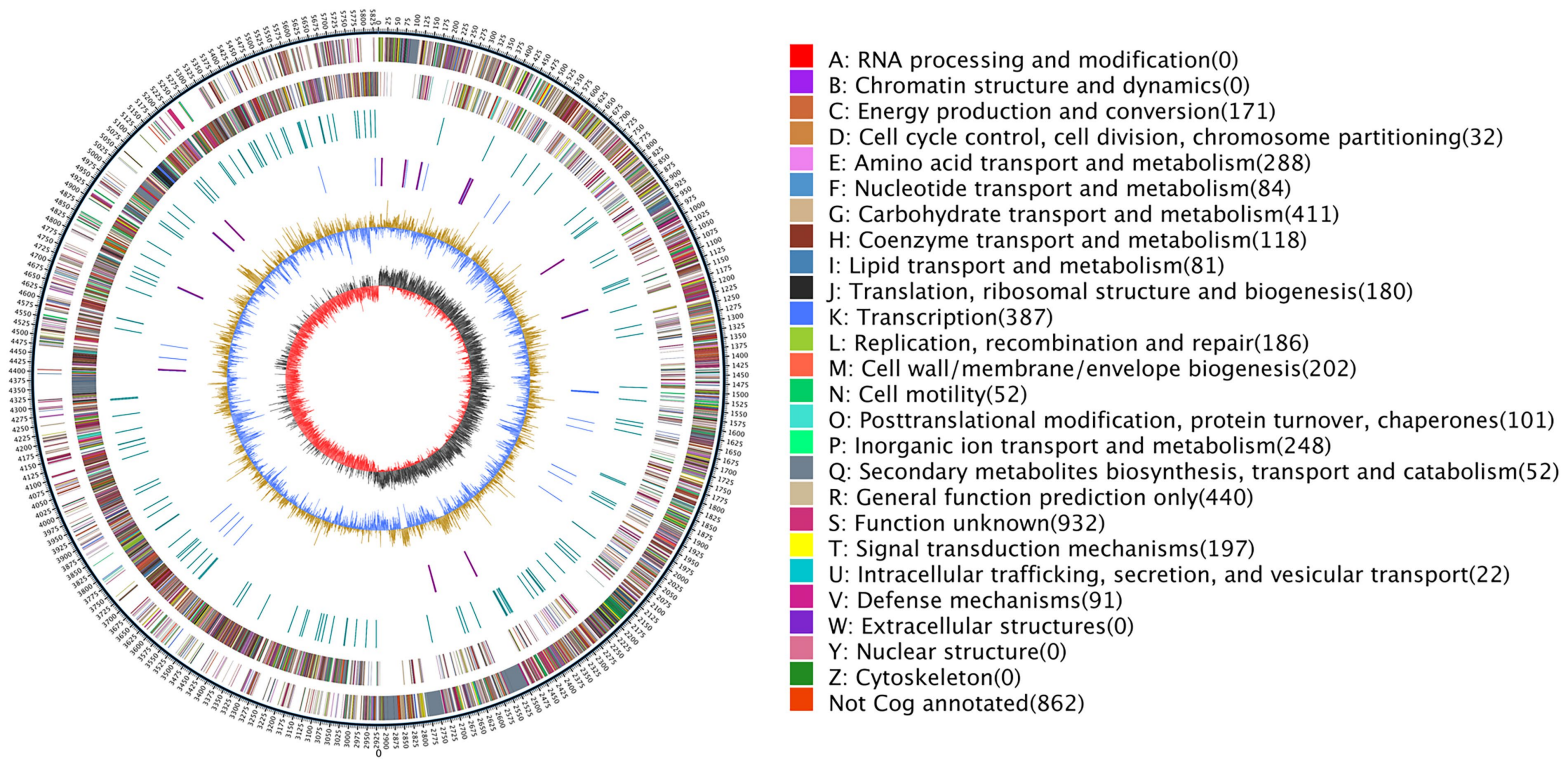
Genome map of *Paenibacillus peoriae* ZBSF16. The distribution of the circle from the outside indicates the genome size, forward CDS, reverse CDS, repeat sequence, tRNA (blue), rRNA (purple), GC ratio (yellow and blue indicate regions where the GC ratio is higher than average and lower than average, respectively), and CG skew positive (dark) and negative (red).

The completed genome of the rod-shaped bacterium *P. peoria* ZBSF16^1^ has been shown to be composed of one circular chromosome of 5,839,239 bp in size, with an average G + C content of 45.62% (Figure 3). The details of the assembly information and genomic features are summarized in Supplementary Tables 3, 4. A total of 5,188 predicted genes were identified in the genome, including 4,944 protein-coding sequences, 39 ribosomal RNA operons, 109 tRNAs, and 4 other RNAs. Genes associated with carbohydrate transport and metabolism (7.98%) were the highest density, followed by transcription (7.51%), amino acid transport and metabolism (5.59%), inorganic ion transport and metabolism (4.81%), signal transduction mechanisms (3.82%), replication, cell wall/membrane/envelope biogenesis (3.92%), replication, recombination, and repair (3.61%) and energy production and conversion (3.31%) (Figure 3). In addition, four crisprs were involved in ZBSF16, and the length of the repeated sequences ranged from 19 to 30 bp (Supplementary Table 4).

### Comparison of the *Paenibacillus peoriae* ZBSF 16 genome with other completely sequenced *Paenibacillus peoriae* strains

#### Phylogenetic tree

**Figure 4:**
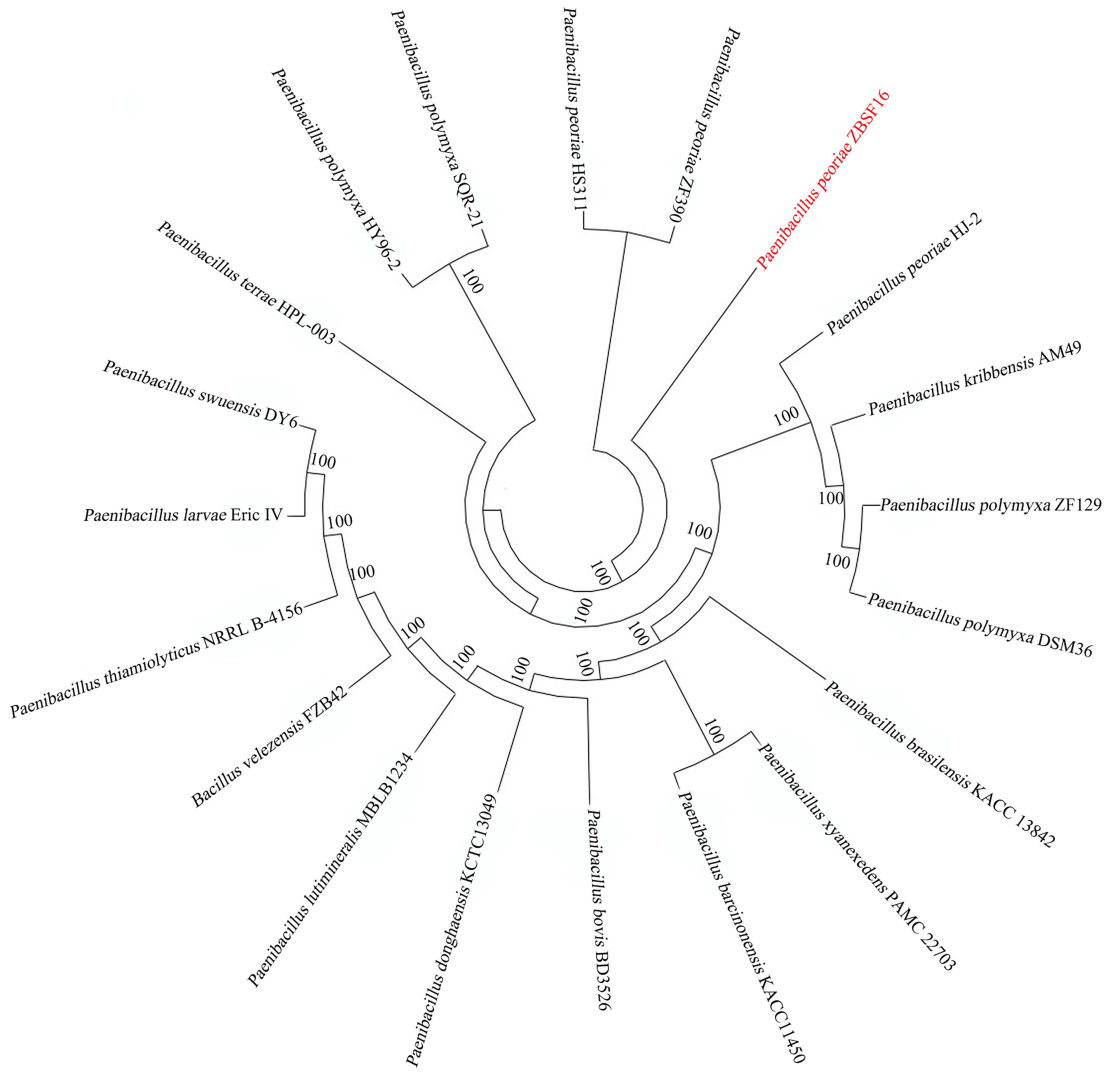
Phylogenetic analysis of *Paenibacillus peoriae* ZBSF16 against six other *Paenibacillus* from genomes using PhyloPhlAn 3.0.2.

To determine the relationships of *P. peoria* ZBSF16 with *Paenibacillus* spp. strains, phylogenetic trees based on the 16S rRNA gene sequences were built. The result indicated that ZBSF16 was close to the strain *P. peoria* ZF390; however, *P. kribbensis* AM49 and *P. peoria* ZF390 were in a clade (Supplementary Figure 4A). Additionally, strain ZBSF16 was clearly classified as *P. peoria* in the phylogenetic tree based on the MLSA, and *P. peoria* ZBSF16 was most closely related to strains *P. peoria* ZF390, *P. peoria* HS311 and *P. peoria* HJ-2 (Supplementary Figure 4B). PhyloPhlAn method was performed to verify the evolutionary position. As expected, *P. peoria* ZBSF16 was most closely related to strains *P. peoria* ZF390, *P. peoria* HS311 (Figure 4).

### ANI and DDH analysis

Average nucleotide identity (ANI) and DNA–DNA hybridization (DDH) are powerful approaches for evolutionary distance assessment between bacteria at the genomic level, and compared strains usually with ANI values > 96% and DDH values ≥ 70% are regarded as the same species (Richter and Rosselló-Móra, 2009; Jiang et al., 2022). ANI values showed that ZBSF16 between *P. peoria* ZF390, HS311 and HJ-2 were 95.22, 95.23 and 95.24%, respectively. However, the DDH value between ZBSF16 and *P. peoria* HS311 was > 70% (Supplementary Figure 4). Obviously, ZBSF16 did not belong to *P. polymyxa* and *P. kribbensis*, according to the lower ANI values (< 91%) and DDH values (< 50%; Supplementary Figure 5).

### Comparison of ZBSF16 with *Paenibacillus peoriae* strains

In comparison, the entire genome size of the four *P. peoriae* strains ranged from 5.84 to 6.19 Mb, the G + C content ranged from 44.99 to 45.62%, and the predicted coding genes ranged from 5,188 to 5,894. Furthermore, the genomes of strains ZF390 and HS311 contained three and one plasmids, respectively. ZBSF16 and HJ-2 both contained one circular chromosome, and the additional genomic features of the six strains are described in Table 1.

**Table 1:** Genomic features of *Paenibacillus peoriae* ZBSF16 and other *P. peoriae* strains.

Features	*P. peoriae* ZBSF16	*P. peoriae* ZF390	*P. peoriae* HJ-2	*P. peoriae* HS311	*P. polymyxa* HY96-2	*P. polymyxa* SQR21	*P. kribbensis* AM49
Size (bp)	5,839,239	6,193,667	6,001,192	6,006,533	5,745,779	5,828,436	5,778,702
GC content (%)	45.62	44.99	45	45.47	45.60	45.60	46.80
Replicons	Chromosome	Chromosome; Plasmid pPlas1; plasmid pPlas2; plasmid pPlas3	Chromosome	Chromosome; plasmid unnamed	chromosome	chromosome	chromosome
Total genes	5,188	5,894	5,439	5,408	4,955	5,128	5,149
Predicted no. of CDS	4,944	5,749	5,237	5,131	4,799	4,974	5,023
Ribosomal RNA	39	40	39	39	42	39	30
Transfer RNA	109	101	108	99	110	111	92
Other RNA	4	4	N/A	1	4	4	4
CRISPR	4	N/A	9	1	N/A	2	4
Pseudogene	92	115	N/A	138	136	78	184

**Figure 5:**
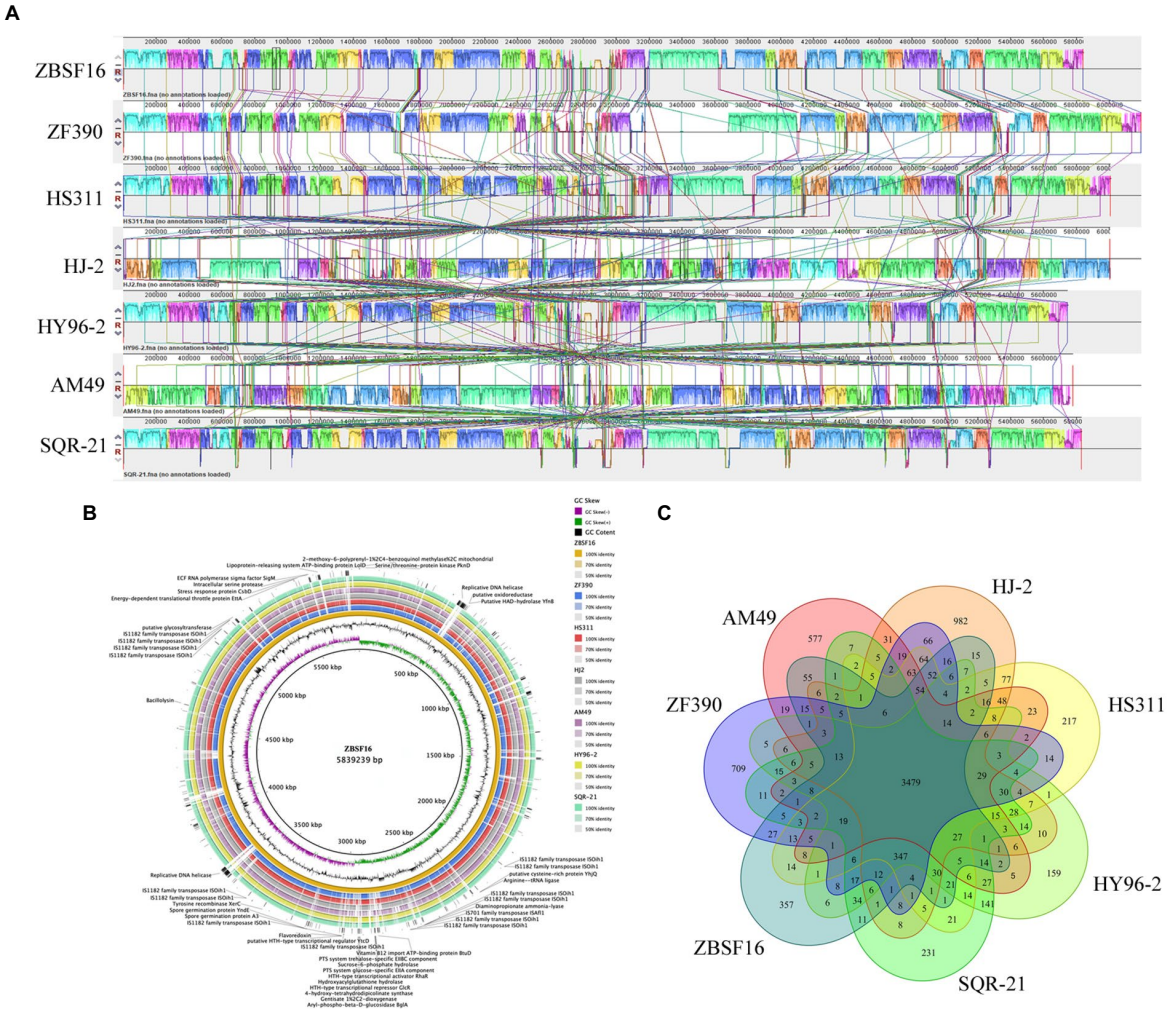
Comparison of *Paenibacillus peoriae* ZBSF16 genome sequences against six other *Paenibacillus* genome sequences. **(A)** Synteny analysis of *P. peoriae* ZBSF16 with the *P. peoriae* ZF390, *P. peoriae* HS311, *P. peoriae* HJ-2, *P. polymyxa* HY96-2 *P. polymyxa* SQR-21 and *P. kribbensis* AM49 genomes. Pairwise alignments of the genomes were generated using MAUVE. The genome of strain ZBSF16 was used as the reference genome. Boxes with the same color indicate syntenic regions. Boxes below the horizontal strain line indicate inverted regions. Rearrangements are shown by colored lines. Scale is in nucleotides. **(B)** Pangenome analysis with closely related strains identified the unique genes present in the query genomes that are highlighted in the outermost circle, the strain ZBSF16 as the query genome is placed in the innermost circle. **(C)** Venn diagram showing the number of clusters of orthologous genes shared and unique genes.

To evaluate the evolutionary distance among these sequenced strains in relation to several *Paenibacillus* strains, the genome sequence of ZBSF16 was compared to three sequenced *P. peoriae* strains (ZF390, HS311 and HJ-2), two *P. polymyxa* strains (HY96-2 and SQR-21) and one *P. kribbensis* (AM49) by mauve. The alignments among *Paenibacillus* strains are presented in Figure 5A. Horizontal gene transfer was obviously observed among *Paenibacillus* strains, and the ZBSF16 genome is much more similar to HS311 than to ZF390 within *P. peoriae* strains based on comparative analysis. There were 3,479 conserved genes shared by the seven sequenced strains of the *Paenibacillus* strains, and 3,960 genes were shared within the four sequenced *P. peoriae* strains, including ZBSF16, ZF390, HS311 and HJ-2. In detail, ZBSF16 shared 4,152, 4,143 and 4,135 genes with ZF390, HS311 and HJ-2, respectively. Furthermore, 357 unique genes were present in the genome of *P. peoriae* ZBSF16, genomes with their unique regions are presented in circular images (Figure 5B), and the functions of most unique genes are still unknown. Notably, only 3,772 genes were shared by ZBSF16 and *P. kribbensis* AM49, which is less than those in The *P. polymyxa* strains (Figures 5B,C; Supplementary Figure 6).

### Genetic basis for promoting plant growth

IAA is an important phytohormone that controls cell enlargement and tissue differentiation in plants. In this study, ZBSF16 showed a higher IAA biosynthetic capacity (28.67 μg/ml; Figure 2A), and 12 genes related to IAA biosynthesis were identified in strain ZBSF16. Nine genes in the IAA biosynthesis pathway were shared among the four *P. peoriae* strains with homology higher than 90%, except for three genes (*trpE*, *trpG* and *trpCF*) that were not found in strain HJ-2 (Table 2). As a major essential nutrient, phosphorus and nitrogen are necessary for the growth and development of plants, and ZBSF16 exhibits the capability of phosphate solubilization and nitrogen fixation (Figure 2D). Additionally, comparative genome analysis showed 14 genes related to phosphate solubilization in ZBSF16, which was highly similar to ZF390, HS311 and HJ-2, and the gene *iap*, which is shared by strain ZF390 and HS311 (Table 2). Furthermore, 15 genes responsible for nitrogen fixation were all found in the genomes of ZBSF16, HS311 and HJ-2, most of which were highly conserved, with sequence identities ranging from 93 to 100%. However, *nifH*, *nifN*, *nifB*, *nifD*, *nifE*, *nifK*, *nifX* and *hesA* were absent in strain ZF390 (Table 2). Meanwhile, 30 genes involved in flagella and 12 genes related to biofilm formation were discovered in strain ZBSF16, and 40 genes involved in flagella (except for *fliD* and *fliS*) and biofilm formation exhibited high conservation (> 88%) in ZF390, HS311, HJ-2 and ZBSF16 (Supplementary Tables 5, 6). Quorum sensing (QS) relegated many traits of bacteria, including biofilm formation and colonization. QS is conserved across hundreds of species belonging to the *Paenibacillaceae* family, and seven genes related to QS were identified in *P. peoriae* strains in this study (Supplementary Table 7). Additionally, 11 genes associated with the chemtaxis and two-component systems (TCS), except *CitG* and *DcuS*, were conservative in different strains of *P. peoriae* (Supplementary Table 8).

**Table 2:** Homolog analysis of genes involved in plant growth promotion in *Paenibacillus peoriae* ZBSF16 and other *P. peoriae* strains.

Genes	Product definition	*P. peoriae* ZBSF16		*P. peoriae* ZF390		*P. peoriae* HS311		*P. peoriae* HJ-2	
		Locus Tag	Protein ID	Protein ID	Homology (%)	Protein ID	Homology (%)	Protein ID	Homology (%)
Indole-3-acetic acid biosynthesis genes									
*acoc*	Chorismite synthase	MLD56_14630	UMY52826.1	WP_007430826.1	99.49	WP_007430826.1	99.49	NA	100.00
*pheB*	Chorismite mutase	MLD56_05715	UMY55941.1	WP_013369718.1	99.17	WP_013369718.1	99.17	NA	99.72
*aroF*	3-Deoxy-7-phosphoheptulonate synthase/chorismite mutase	MLD56_07785	UMY56321.1	WP_017426658.1	97.97	WP_013309435.1	99.71	NA	99.71
	Tryptophan-rich sensory protein	MLD56_02865	UMY55416.1	WP_014279544.1	89.64	WP_014279544.1	89.64	NA	96.02
*trpA*	Tryptophan synthase subunit alpha	MLD56_14590	UMY52818.1	WP_013310712.1	98.13	WP_013310712.1	98.13	NA	98.51
*trpB*	Tryptophan synthase subunit beta	MLD56_14595	UMY52819.1	WP_014282083.1	96.73	WP_014282083.1	96.73	NA	99.25
*trpS*	Tryptophan--tRNA ligase	MLD56_20860	UMY53981.1	WP_016819987.1	98.18	WP_013311859.1	97.26	NA	96.66
*trpC*	Indole-3-glycerol phosphate synthase TrpC	MLD56_14605	UMY52821.1	WP_017427551.1	94.30	WP_013371667.1	95.06	NA	97.35
*trpD*	Anthranilate phosphoribosyltransferase	MLD56_14610	UMY52822.1	WP_013371668.1	95.98	WP_013371668.1	95.98	NA	97.70
*trpE*	Anthranilate synthase component I	MLD56_14615	UMY52823.1	WP_007430823.1	97.87	WP_007430823.1	97.87	NA	NA
*trpG*	Glutamine amidotransferase	MLD56_18435	UMY53538.1	WP_007431477.1	91.83	WP_007431477.1	91.83	NA	NA
*trpCF*	Phosphoribosylanthranilate isomerase	MLD56_14600	UMY52820.1	WP_019687860.1	91.23	WP_019687860.1	91.23	NA	NA
*ipdC*	Thiamine pyrophosphate-binding protein	MLD56_00395	UMY54998.1	WP_007428062.1	100.00	WP_007428062.1	100.00	NA	98.75
Phosphate solubilization genes									
*phoN*	Phosphatase PAP2 family protein	MLD56_05880	UMY55971.1	WP_010347599.1	87.07	WP_013309103.1	97.79	NA	99.97
*iap*	Aminopeptidase	NA	NA	WP_013309451.1	NA	WP_013309451.1	NA	NA	NA
*phoA*	Alkaline phosphatase	MLD56_07175	UMY56211.1	WP_019686611.	93.21	WP_013309329.1	97.48	NA	97.03
*phnE*	Phosphonate ABC transporter, permease protein PhnE	MLD56_21880	UMY54161.1	WP_016324733.1	99.30	WP_016324733.1	99.30	NA	99.30
*phnE*	Phosphonate ABC transporter, permease protein PhnE	MLD56_21885	UMY54162.1	WP_016820374.1	98.87	WP_016820374.1	98.87	NA	99.25
*phnD*	Phosphonate ABC transporter substrate-binding protein	MLD56_21870	UMY54159.1	WP_010344588.1	96.89	WP_010344588.1	96.89	NA	99.69
*phnC*	Phosphonate ABC transporter ATP-binding protein	MLD56_21875	UMY54160.1	WP_020723499.1	98.83	WP_020723499.1	98.83	NA	97.66
*pstS*	Phosphate ABC transporter substrate-binding protein PstS	MLD56_08410	UMY56438.1	WP_016819622.1	98.70	WP_016819622.1	98.70	NA	99.35
*pstC*	Phosphate ABC transporter permease PstC	MLD56_08415	UMY56439.1	WP_013370343.1	99.66	WP_053325097.1	99.33	NA	99.68
*pstA*	Phosphate ABC transporter permease PstA	MLD56_08420	UMY57301.1	WP_013309592.1	99.66	WP_013309592.1	99.66	NA	100
*pstB*	Phosphate ABC transporter ATP-binding protein PstB	MLD56_08425	UMY56440.1	WP_013370344.1	97.86	WP_013370344.1	97.86	NA	99.29
*pstB*	Phosphate ABC transporter ATP-binding protein PstB	MLD56_08490	UMY56453.1	WP_007429703.1	96.83	WP_007429703.1	96.83	NA	99.21
*phoU*	Phosphate signaling complex protein PhoU	MLD56_08495	UMY56454.1	WP_016819636.1	96.80	WP_016819636.1	96.80	NA	100
*phoN*	Phosphatase PAP2 family protein	MLD56_05880	UMY55971.1	WP_010347599.1	87.07	WP_013309103.1	97.79	NA	99.97
Nitrate transport and nitrate/nitrite reduction									
*narI*	Nitrate reductase gamma subunit	MLD56_17955	UMY57358.1	WP_013372381.1	95.59	WP_013311337.1	96.93	NA	96.37
*narJ*	Nitrate reductase molybdenum cofactor assembly chaperone	MLD56_17960	UMY57359.1	WP_010345152.1	95.72	WP_010345152.1	95.72	NA	98.40
*narH*	Nitrate reductase beta subunit	MLD56_17965	UMY53450.1	WP_016324613.1	92.25	WP_014282714.1	99.43	NA	99.62
*narG*	Nitrate reductase alpha subunit	MLD56_17970	UMY53451.1	WP_007431447.1	93.95	WP_007431447.1	93.95	NA	98.62
*narK*	MFS transporter NNP family nitrate/nitrite transporter	MLD56_17930	UMY53446.1	WP_013311332.1	98.18	WP_013311332.1	98.18	NA	98.63
Niterate transport and reduction									
*nirD*	Nitrite reductase small subunit NirD	MLD56_03440	UMY55525.1	WP_017428677.1	93.58	WP_017428677.1	93.58	NA	96.33
*nirC*	Nitrite transporter NirC	MLD56_04985	UMY55818.1	WP_016819917.1	98.47	WP_016819917.1	98.47	NA	90.46
*nirB*	Nitrite reductase large subunit NirB	MLD56_03435	UMY55524.1	WP_016818403.1	97.65	WP_016818403.1	97.65	NA	98.27
*amtB*	Ammonium transporter Amt family	MLD56_09035	UMY56554.1	WP_010348916.1	96.79	WP_007429827.1	97.00	NA	99.79
*nifH*	Nitrogenase iron protein NifH	MLD56_05440	UMY55888.1	NA	NA	WP_007429042.1	98.26	NA	100
*nifN*	Nitrogenase molybdenum-iron protein NifN	MLD56_05460	UMY55892.1	NA	NA	WP_014280100.1	98.16	NA	93.08
*nifB*	Nitrogenase fixation protein NifB	MLD56_05435	UMY55887.1	NA	NA	WP_014280095.1	95.79	NA	97.35
*nifD*	Nitrogenase fixation protein NifD	MLD56_05445	UMY55889.1	NA	NA	WP_007429043.1	97.93	NA	95.69
*nifU*	Nitrogenase fixation protein NifU	MLD56_21125	UMY54031.1	WP_013373004.1	100	WP_013373004.1	100	NA	100
*nifE*	Nitrogenase molybdenum-cofator synthesis protein NifE	MLD56_05455	UMY55891.1	NA	NA	WP_014280099.1	96.91	NA	99.56
*nifK*	Nitrogenase molybdenum-iron protein subunit beta	MLD56_RS05450	UMY55890.1	NA	NA	WP_007429044.1	97.45	NA	97.45
*nifX*	Nitrogen fixation protein NifX	MLD56_RS05465	UMY55893.1	NA	NA	WP_014280101.1	97.67	NA	96.90
*hesA*	HesA/MoeB/ThiF family protein	MLD56_RS05470	UMY55894.1	NA	NA	WP_014280102.1	100.00	NA	97.24

NA, not available.

### Genes/gene cluster for antibiotic synthesis and induction of resistance

**Figure 6:**
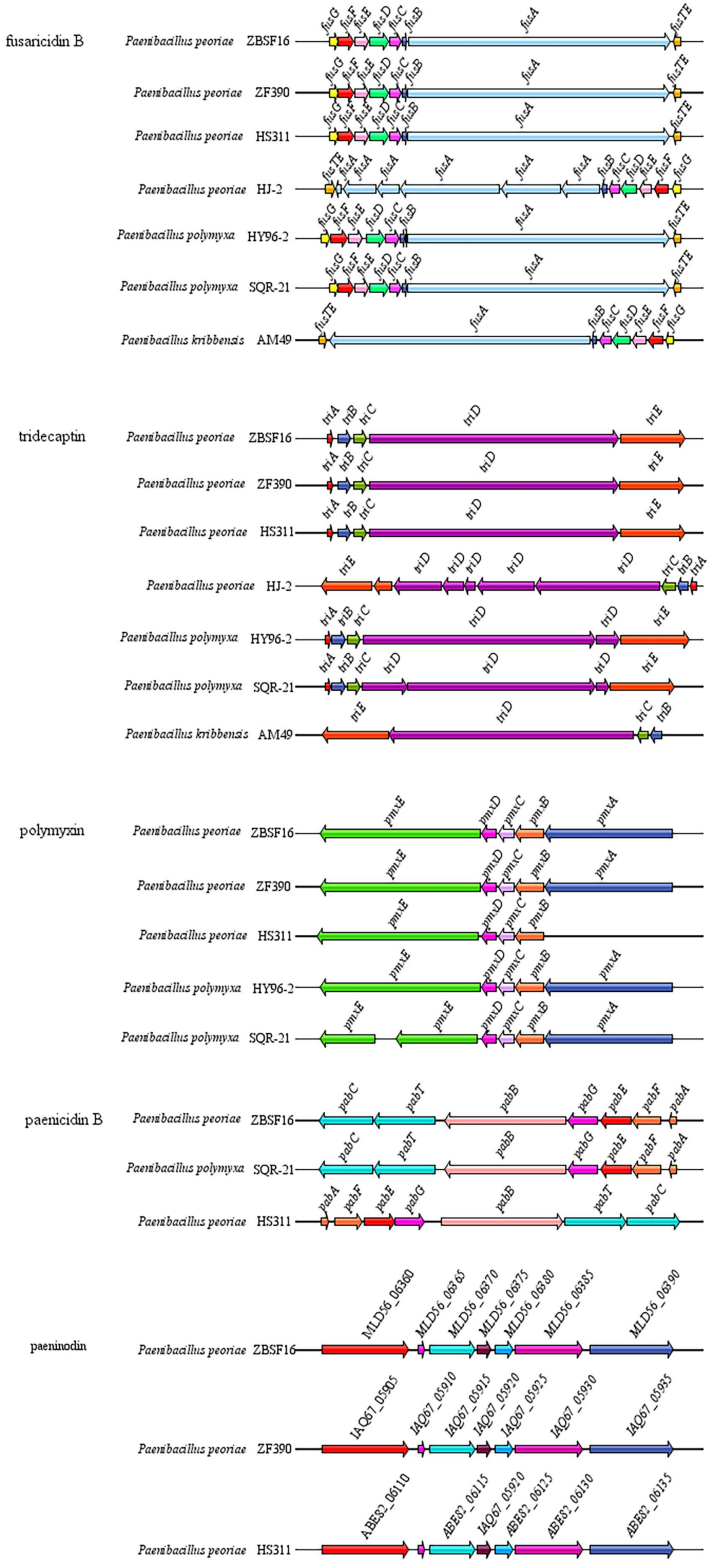
Comparison of antibiotic synthesis clusters of *Paenibacillus* strains. Antibiotic synthesis clusters were identified using antiSMASH, and gene cluster intraspecific genes were compared.

*P. peoriae* ZBSF16 showed potent broad-spectrum antifungal activities. Based on the antiSMASH database, 14 clusters related to secondary metabolite synthesis were identified in ZBSF16. Among these gene clusters, three clusters (Cluster 1 related to fusarcidinB, Cluster 8 related to cyclic-lactone-autoinducer, and Cluster 9 related to tridecaptin) were shared among the four *P. peoriae* strains, the two *P. polymyxa* and *P. kribbensis*; the functions of fusarcidin B and tridecaptin were antifungal and antibacterial, respectively. Cluster 3 related to paenibacillin was specific and only found in strain ZBSF16, which was a kind of lantibiotic. In addition, polymyxin and paenilan did not appear in *P. kribbensis*, paeninodin could not be detected in *P. polymyxa*, and genes related to Cluster 17 encoding the biosynthesis of paenilan, pelgipeptin, aurantinin and so on were not found in ZBSF16 (Figure 6; Supplementary Table 9).

The resistance inducer biosynthesis gene cluster, including 11 genes related to ISR and 3 genes involved in PAMP-triggered immunity (PTI), was analyzed in strain ZBSF16, which is highly conserved in the selected *P. peoriae* strains (> 79% identity). The genes *alsS* and *budA* were identified in strain ZBSF16, which showed a lower similarity to ZF390. The gene *flgL* involved in PTI of plants showed higher similarity to ZF390, and it could not be identified in strains HS311 and HJ-2 (Table 3).

**Table 3:** Genes related to synthesis resistance inducer in *Paenibacillus peoriae* ZBSF16 and other *P. peoriae* strains.

Genes	Resistance inducers	Plant resistance type	Product definition	*P. peoriae* ZBSF16		*P. peoriae* ZF390		*P. peoriae* HS311		*P. peoriae* HJ-2	
				Locus tag	Protein ID	Protein ID	Homology (%)	Protein ID	Homology (%)	Protein ID	Homology (%)
*alsS*	2,3-Butanediol	ISR	Acetolactate synthase	MLD56_10755	UMY56883.1	WP_003206007.1	75.92	WP_013310040.1	95.43	NA	95.96
*budA/ alsD*	2,3-Butanediol	ISR	Acetolactate decarboxlase	MLD56_10750	UMY56882.1	WP_000215036.1	68.25	WP_016821069.1	97.18	NA	97.58
*bdh*	2,3-Butanediol	ISR	2，3-Butanediol dehydrogenase	MLD56_18150	UMY53485.1	WP_019688213.1	98.29	WP_013311373.1	99.43	NA	96.00
*ilvN*	2,3-Butanediol	ISR	Acetolactate synthase small subunit	MLD56_07545	UMY56280.1	WP_007429525.1	98.76	WP_013309386.1	99.38	NA	99.38
*metH*	Methanethio	ISR	Methionine synthase	MLD56_13735	UMY52659.1	WP_010345928.1	96.68	WP_010345928.1	96.68	NA	80.94
*metE*	Methanethio	ISR	5-Methyltetrahydro- pteroyltriglutamate- homocysteine S-methyltransferase	MLD56_24010	UMY54554.1	WP_013373554.1	93.47	WP_013312443.1	97.73	NA	96.50
*ispF*	Isoprene	ISR	2-C-methyl-D-erythritol 2,4-cyclodiphosp- hata	MLD56_22685	UMY54300.1	WP_000488386.1	100	WP_007432605.1	98.10	NA	98.73
*ispE*	Isoprene	ISR	4-(cytidine 5′-diphospho)-2-C- methyl-D-erythritol kinase	MLD56_00170	UMY54955.1	WP_013308121.1	99.65	WP_013308121.1	99.65	NA	98.94
*gcpE*	Isoprene	ISR	Flavodoxin- dependent (E)-4-hydroxy-3- methylbut-2-enyl- diphosphate synthae	MLD56_19660	UMY53756.1	WP_010348073.1	98.92	WP_010348073.1	98.92	NA	100
*lytB*	Isoprene	ISR	4-hydroxy-3 -methylbut-2-enyl diphosphate reductase	MLD56_07780	UMY56320.1	WP_013309434.1	99.37	WP_013309434.1	99.37	NA	99.00
*fni*	Isoprene	ISR	Type 2 isopentenyl- diphosphate Delta-isomerase	MLD56_23495	UMY54455.1	WP_017427145.1	91.80	WP_013312347.1	96.45	NA	96.72
*guaB*	Peptidoglycan	PTI	carboxypeptidase	MLD56_00435	UMY55002.1	WP_017427215.1	97.94	WP_017427215.1	97.94	NA	97.94
*flgL*	*Flagenllin*	PTI	flagellin	MLD56_23175	UMY54392.1	WP_016822919.1	96.44	N/A	N/A	NA	NA
*tuf*	*EF-Tu*	PTI	Elongation factor Tu	MLD56_22575	UMY54278.1	WP_017815361.1	96.21	WP_017815361.1	96.21	NA	98.99

NA, not available.

## Discussion

*Paenibacillus* is widely distributed in a variety of environments, including wetlands, meadow soil, desert sand, oceans, wheat soil rhizospheres, cucumber greenhouses and infected honeybees (Jeon et al., 2009; Wang et al., 2013; Ahn et al., 2014). The genus *Paenibacillus* is reported to have the ability to promote the growth of many plants, such as maize, wheat, tomato, and pumpkin (Hao and Chen, 2017; Dixit et al., 2018). The genome size of *Paenibacillus* species ranges from 3.02 Mbp to 8.82 Mbp. As a member of 200 species in *Paenibacillus*, *P. peoriae* was described to play a role in promoting the growth of plants by some studies in the past and was confirmed in this study (Figure 2), with a genome size of 5.74–6.19 Mbp and GC content of 44.99–45.62% (Table 1). *P. peoriae* was close to *P. polymyxa* and *P. kribbensis* in terms of evolutionary status, and ZBSF16 was identified and confirmed to belong to *P. peoriae* by ANI and DDH. Compared to *P. peoriae* HJ-2, which presented antagonistic activity against *Fusarium* spp., ZBSF16 had a broad antifungal and antibacterial spectrum, which could protect against 10 species of fungi and 2 species of bacteria.

Many PGPRs, including *Bacillus*, *Rahnella*, *Pseudomonas*, *Klebsiella*, *Agrobacterium* and *Paenibacillus* sp. can produce IAA to stimulate the growth of plants, and *Paenibacillus* nonsymbiotic bacteria yielded high concentrations of IAA (in the range of 4.90–0.19 IAA/mg biomass; Shokri and Emtiazi, 2010; Trinh et al., 2018). *P. polymyxa*, *P. borealis*, and *P. terrae* showed the secretion of a significant amount of IAA, but no *P. graminis* had the ability to produce IAA (Navarro-Noya et al., 2012; Kim et al., 2017). *P. peoriae* HJ-2 isolated from soil significantly promoted the growth of *P. polyphylla*, and *P. peoriae* ZBSF16 for the first time was used to describe the ability to synthesize IAA and promote the growth of grape, with IAA production of 28.67 μg ml^−1^. The various pathways for IAA biosynthesis include tryptophan (Trp), tryptamine (Tam), indole-3-pyruvic acid (IPyA) and indole-3-acetamide (IAAm) pathways, and the IPyA pathway was suggested in *Paenibacillus* because of the absence of tryptophan monooxygenase or indole-3-acetamide hydrolase (Mano and Nemoto, 2012; Xie et al., 2016). In addition, the *ipdC* gene, encoding a key enzyme in the IPyA pathway, is shared in all *Paenibacillus* (Xie et al., 2016). In this study, *ipdC* homologies were present in all sequenced *P. peoriae*, which demonstrated that *P. peoriae* may rely on the IPyA pathway for IAA synthesis.

*P. polymyxa* strains have long been known to solubilize phosphate, which carries the *phn* genes (*phnABCDEWXM*) responsible for solubilizing organic phosphate (Zhou et al., 2020; Soni et al., 2021). The *phnB* gene was absent in some species of *Paenibacillus*, including *P. beijingensis* 1–18, *P. peoriae* KCTC 3763 and *P. terrae* HPL-003 (Jeong et al., 2012; Shin et al., 2012; Li L. et al., 2019). In this study, phnA and phnB were not found in the genomes of *P. peoriae*. The Pst (phosphate-specific transport) system is a major transport system for Pi. The *pst* operon of *Paenibacillu* is composed of *pstS*, *pstC*, *pstA* and *pstB* (Li et al., 2020), and the four *pst* genes were all present in *P. peoriae* ZBSF16, which contribute to the solubilization of phosphate. It has been reported that *Rahnella aquatilis* ZF7 can produce acid, which may have high activity for solubilizing organic phosphate (Yuan et al., 2020). A higher phosphate solubilization ability of *P. peoriae* ZBSF16 was observed, although the pH value of ZBSF16 remained alkaline when cultured.

Nitrogen fixation is one characteristic of the genus *Paenibacillus*, and more than 20 species of the genus *Paenibacillus* can fix nitrogen (Grady et al., 2016; He et al., 2021). Nitrogen fixation is mainly catalyzed by Mo-nitrogenase, and the *nif* gene cluster (*nif*B, *nif*H, *nif*D, *nif*K, *nif*E, *nif*N, *nif*X, *hes*A and *nif*V) encoding Mo-nitrogenase is shared in N_2_-fixing *Paenibacillus* strains (Xie et al., 2014). When the nif gene cluster is lost, non-N_2_-fixing strains are produced, such as *P. peoriae* KTCT 3763, *P. polymyxa* SC2 and *P. polymyxa* E681 (Kim et al., 2010; Ma et al., 2011). When acquiring the *vnf* and *anf* genes, strains of *vnf*HDGKEN encoding V-nitrogenase and *anf*HDGK encoding Fe-nitrogenase appeared, such as *P. azotofixans* ATCC 35681 and *P. forsythia* T98 (Xie et al., 2014, 2016). Most likely due to gene loss, the *nif*V gene was absent in the gene cluster in *P. peoriae* ZBSF16, but ZBSF16 retained its nitrogen-fixing capacity.

The genus *Paenibacillus* is known for its ability to produce antibacterial metabolites, including fusaricidins, pelgipeptin, surfactins and polymyxins (Grady et al., 2016). The antibacterial metabolites of *P. polymyxa* ZF129 and *P. polymyxa* ZF197 were significantly different, but paeninodin, fusaricidin, paenibacterin and tridecaptin were shared by the two strains (Li et al., 2020). In our study, fusaricidin B, tridecaptin, polymyxin and paenicidin B were found in *P. peoriae* ZBSF16, which contribute to its strong antipathogenic activities. In addition, fusaricidin B, tridecaptin and polymyxin were conserved in *P. peoriae*, *P. polymyxa* and *P. kibbensis*, which were also shared in *P. polymyxa* ZF129 and *P. polymyxa* ZF197. The antifungal mechanism of fusaricidin is permeabilization and disruption of cell membranes (Jiang et al., 2022), which may be one of the reasons why *P. peoriae* ZBSF16 showed a broad antifungal spectrum.

ISR is the form of induced resistance wherein plant defenses are preconditioned by prior treatment that results in resistance against subsequent challenge by a pathogen or parasite (Choudhary et al., 2007). ISR can increase systemic levels of the plant hormone salicylic acid (SA) and trigger the jasmonic acid/ethylene pathway. *Paenibacillus*-mediated ISR has been demonstrated against fungi (e.g., *C. truncatum*, *C. orbiculare* and *F. oxysporum*) and bacteria (e.g., *Xanthomonas axonopodis* pv. *vesicatoria*, *Erwinia carotovora* subsp. *carotovora*) in pepper, cucumber, banana, and *Arabidopsis thaliana* (Sang et al., 2014; Nakkeeran et al., 2021; Yadav M. et al., 2021). Nine genes involved in ISR were explored in *P. polymyxa*, with higher sequence identity (> 95%) in different strains, while key genes associated with volatile organic compounds (2,3-butanediol, methanethiol and isoprene) were contained (Li et al., 2020). A total of 12 genes related to ISR were found in *P. peoriae* ZBSF16, which were highly similar to those in *P. polymyxa* (homology > 99%). The results demonstrated that *P. peoriae* and *P. polymyxa* could induce similar systemic resistance in plants.

## Conclusion

*P. peoriae* ZBSF16 showed broad-spectrum antagonistic activities against 12 plant pathogens and exhibited obvious biocontrol effects against grape white rot disease. The aim of this study was to reveal the plant growth-promoting and biocontrol mechanisms of *P. peoriae*. Whole-genome analysis and phylogenetic analysis revealed that ZBSF16 belongs to *P. peoriae* and is closely related to *P. peoriae* ZF390. Comparative analysis of the genome of *P. peoriae* ZBSF16 with other *Paenibacillus* spp. indicated that ZBSF16 harbored many genes related to IAA production, nitrogen fixation, phosphate solubilization, biofilms and flagella, which have been proven to be beneficial to plant growth. In addition, genes associated with antibiotic synthesis and induction of resistance were identified. Overall, the features of *P. peoriae* ZBSF16 make it a high-probability biocontrol agent and biofertilizer, and these results will contribute to in-depth research on the mechanisms of plant growth promotion and biocontrol.

## Data availability statement

The datasets presented in this study can be found in online repositories. The names of the repository/repositories and accession number(s) can be found at: NCBI GenBank - CP092831.1.

## Author contributions

LY, YW, and XY conceived and designed the experiments. XY, HJ, TL, PL, and XJ performed the experiments and analyzed the data. LY and YW wrote the manuscript. TL, XJ, PL, and HJ revised the manuscript. All authors contributed to the article and approved the submitted version.

## Funding

This research was supported by Shandong Provincial Natural Science Foundation (ZR2021QC131), Innovation Project of Shandong Academy of Agricultural Sciences (CXGC2022E15), and Shandong Academy of Grape Guide Fund (SDAG2021B06, SDAG2021B10, and SDAG2021B02).

## Conflict of interest

The authors declare that the research was conducted in the absence of any commercial or financial relationships that could be construed as a potential conflict of interest.

## Publisher’s note

All claims expressed in this article are solely those of the authors and do not necessarily represent those of their affiliated organizations, or those of the publisher, the editors and the reviewers. Any product that may be evaluated in this article, or claim that may be made by its manufacturer, is not guaranteed or endorsed by the publisher.

## Supplementary material

The Supplementary material for this article can be found online at: https://www.frontiersin.org/articles/10.3389/fmicb.2022.975344/full#supplementary-material

SUPPLEMENTARY FIGURE 1 General characteristics of *Paenibacillus peoriae* ZBSF16. **(A)** Image of ZBSF16 colony morphology. **(B)** Image of ZBSF16 cells *via* scanning electron microscopy. **(C)** Growth dynamics and pH change of *P. peoriae* ZBSF16. Bars plot the means ± standard deviation of three replicate experiments. P **(D)** Production of protease. **(E)** Cellulose degradation. **(F)** Production of lipase. Determination of NaCl **(G)** and pH **(H)** tolerance capabilities of *P. peoriae* ZBSF16.

SUPPLEMENTARY FIGURE 2 Antagonistic assay and biocontrol effect of *Paenibacillus peoriae* ZBSF16. **(A)** Colony radius and inhibition rate of each microorganism. Bars plot the means ± standard deviation of three replicate experiments. *Coniella vitis* (CV). *Gloeosporium fructigrum* (GF). *Pestalotiopsis clavispora* (Pc). *Alternaria viticola* (Av). *Diaporthe eres* (DE). *Fusarium oxysporum* (Fo). *Botrytis cinerea* (BC)*. Botryosphaeria dothidea* (BD). *Aspergillus niger* (AN). *Fusarium graminearum* (FG). *Fusarium pseudograminearum* (FP). *Allorhizobium vitis* (ALV). **(B,C)** Incidence, disease index and control efficiency of *P. peoriae* ZBSF16. (a1, b1) Inoculated with *C. vitis*; (a2, b2) LB broth; (a3, b3) sterile water; (a4, b4) culture of ZBSF16; (a5, b5) inoculated with *C. vitis* 24 h after inoculation with the culture of ZBSF16; (a6, b6) inoculated culture of ZBSF16 24 h after inoculation with *C. vitis*. **(D)** Disease symptoms and growth state of *Vitis vinifera* (cv. Red globe) inoculated with strain ZBSF16. **(E)** The infection rate and disease index of grape white rot on *Vitis vinifera* (cv. Red globe) inoculated with strain ZBSF16. CK plants were treated with sterile water. Different letters above the bars denote a significant difference at *p* < 0.05 according to Duncan’s multi-range test.

SUPPLEMENTARY FIGURE 3 Determination of antibiotic resistance of *Paenibacillus peoriae* ZBSF16. **(A)** Survival of *P. peoriae* ZBSF16 treated with different antibiotics. Spectinomycin (Spe), streptomycin (Str), ampicillin (Amp), vancomycin (Van), kanamycin (Kan), gentamycin (Gen), chloramphenicol (Chl), tetracycline (Tet) and rifampicin (Rif). **(B)** Minimum inhibitory concentration (MIC) of spectinomycin for strain ZBSF16. **(C)** Minimum bactericidal concentration (MBC) of spectinomycin for strain ZBSF16. **(D)** Hemolysis assay of ZBSF16. **(E)** Siderophores production of *P. peoriae* ZBSF16. **(F)** Population dynamics of *P. peoriae* ZBSF16 in the rhizosphere soil of grape.

SUPPLEMENTARY FIGURE 4 **(A)** Phylogenetic tree for *P. peoriae* ZBSF16 and the genus *Paenibacillus* based on 16S rRNA (*Bacillus velezensis* FZB42 was used as an outgroup). **(B)** Phylogenetic tree of *Paenibacillus peoriae* ZBSF16 among other *Paenibacillus* species. The phylogenetic tree was constructed based on five housekeeping genes (16S rRNA, *gyrB*, *rpoD*, *rho*, and *pgk*) according to the aligned gene sequences using the maximum likelihood method in MEGA 6.0. Bootstrap values (1,000 replicates) are shown at the branch points. The scale bar indicates 0.05 nucleotide substitutions per nucleotide position. GenBank accession numbers associated with the housekeeping loci of all strains can be found in Supplementary Table 1.

SUPPLEMENTARY FIGURE 5 ANI **(A)** and DDH **(B)** value matrix heatmap between *Paenibacillus peoriae* ZBSF16 and six other *Paenibacillus* genome sequences.

SUPPLEMENTARY FIGURE 6 Venn diagram showing the number of clusters of orthologous genes shared and unique genes.

## Footnotes

^1^ https://www.ncbi.nlm.nih.gov/nuccore/CP092831.1
