# Supplementary figures and images for: Comparative genomic and functional analyses of Paenibacillus peoriae ZBSF16 with biocontrol potential against grapevine diseases, provide insights into its genes related to plant growth-promoting and biocontrol mechanisms
Source: Front Microbiol. 2022 Sep 8;13:975344. doi: 10.3389/fmicb.2022.975344 (PMC9492885; doi:10.3389/fmicb.2022.975344)

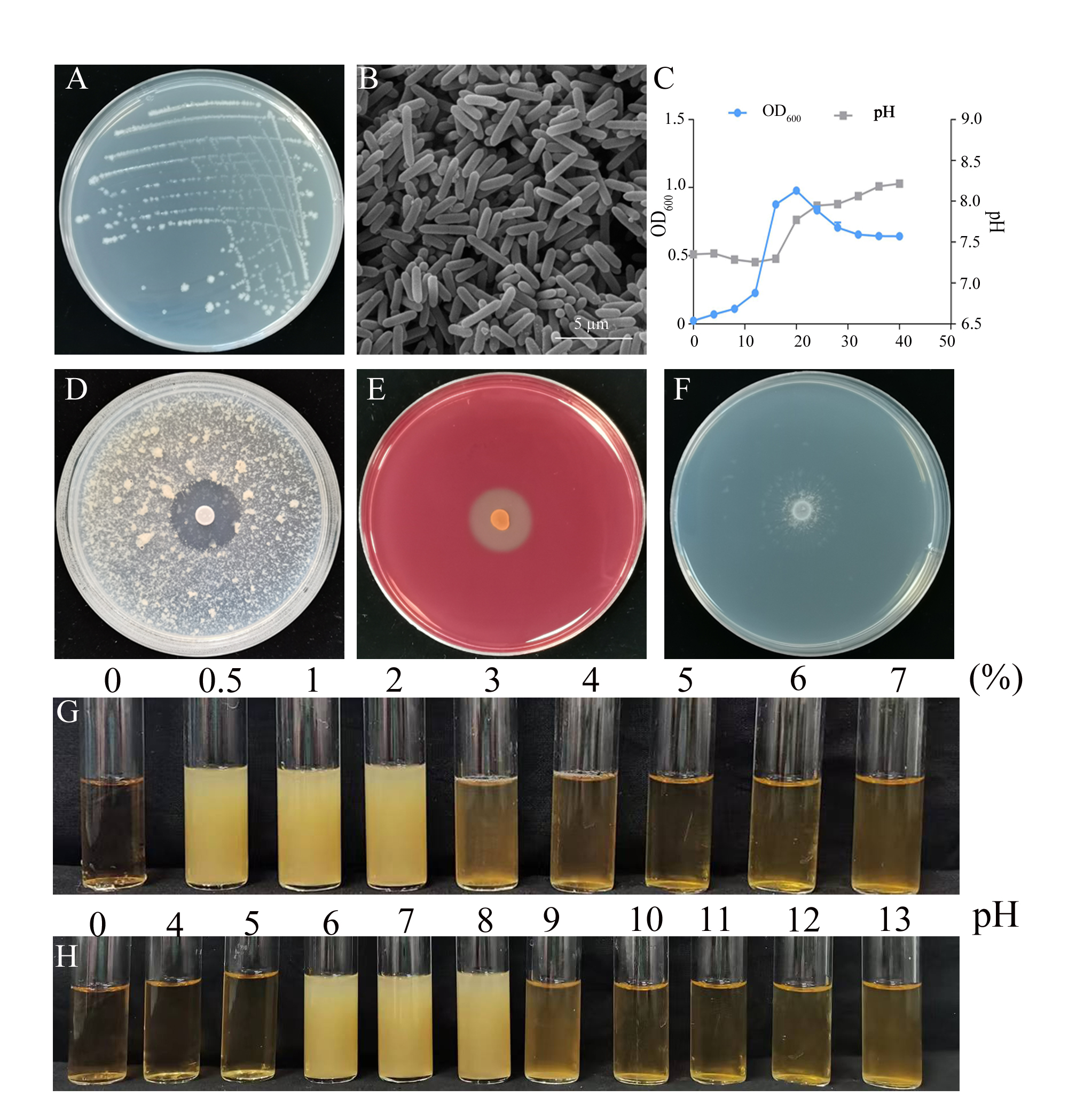

Supplement: SUPPLEMENTARY FIGURE 1 — General characteristics of Paenibacillus peoriae ZBSF16. (A) Image of ZBSF16 colony morphology. (B) Image of ZBSF16 cells via scanning electron microscopy. (C) Growth dynamics and pH change of P. peoriae ZBSF16. Bars plot the means ± standard deviation of three replicate experiments. P (D) Production of protease. (E) Cellulose degradation. (F) Production of lipase. Determination of NaCl (G) and pH (H) tolerance capabilities of P. peoriae ZBSF16. [file Image_1.jpeg]

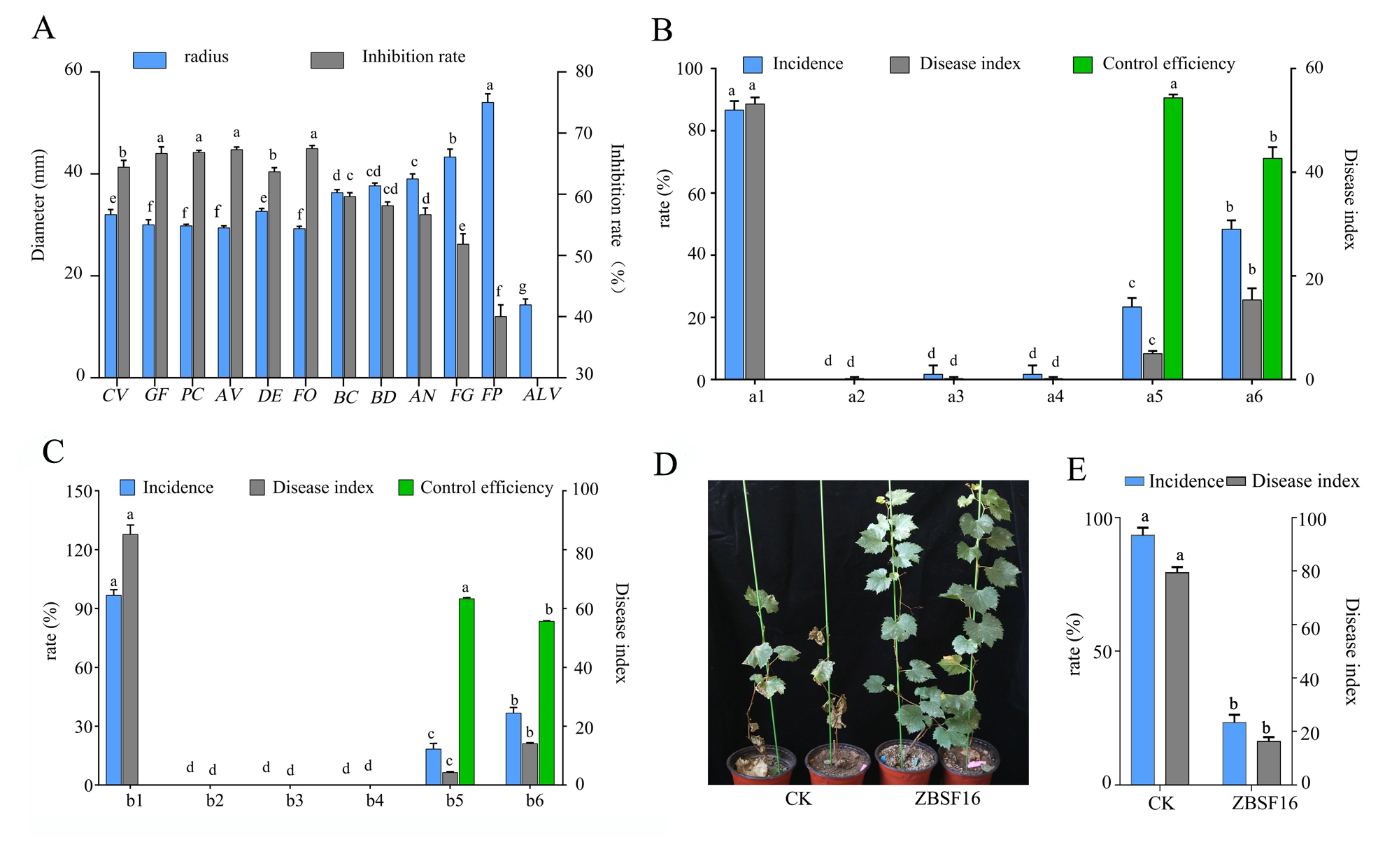

Supplement: SUPPLEMENTARY FIGURE 2 — Antagonistic assay and biocontrol effect of Paenibacillus peoriae ZBSF16. (A) Colony radius and inhibition rate of each microorganism. Bars plot the means ± standard deviation of three replicate experiments. Coniella vitis (CV). Gloeosporium fructigrum (GF). Pestalotiopsis clavispora (Pc). Alternaria viticola (Av). Diaporthe eres (DE). Fusarium oxysporum (Fo). Botrytis cinerea (BC). Botryosphaeria dothidea (BD). Aspergillus niger (AN). Fusarium graminearum (FG). Fusarium pseudograminearum (FP). Allorhizobium vitis (ALV). (B,C) Incidence, disease index and control efficiency of P. peoriae ZBSF16. (a1, b1) Inoculated with C. vitis; (a2, b2) LB broth; (a3, b3) sterile water; (a4, b4) culture of ZBSF16; (a5, b5) inoculated with C. vitis 24 h after inoculation with the culture of ZBSF16; (a6, b6) inoculated culture of ZBSF16 24 h after inoculation with C. vitis. (D) Disease symptoms and growth state of Vitis vinifera (cv. Red globe) inoculated with strain ZBSF16. (E) The infection rate and disease index of grape white rot on Vitis vinifera (cv. Red globe) inoculated with strain ZBSF16. CK plants were treated with sterile water. Different letters above the bars denote a significant difference at p < 0.05 according to Duncan’s multi-range test. [file Image_2.jpeg]

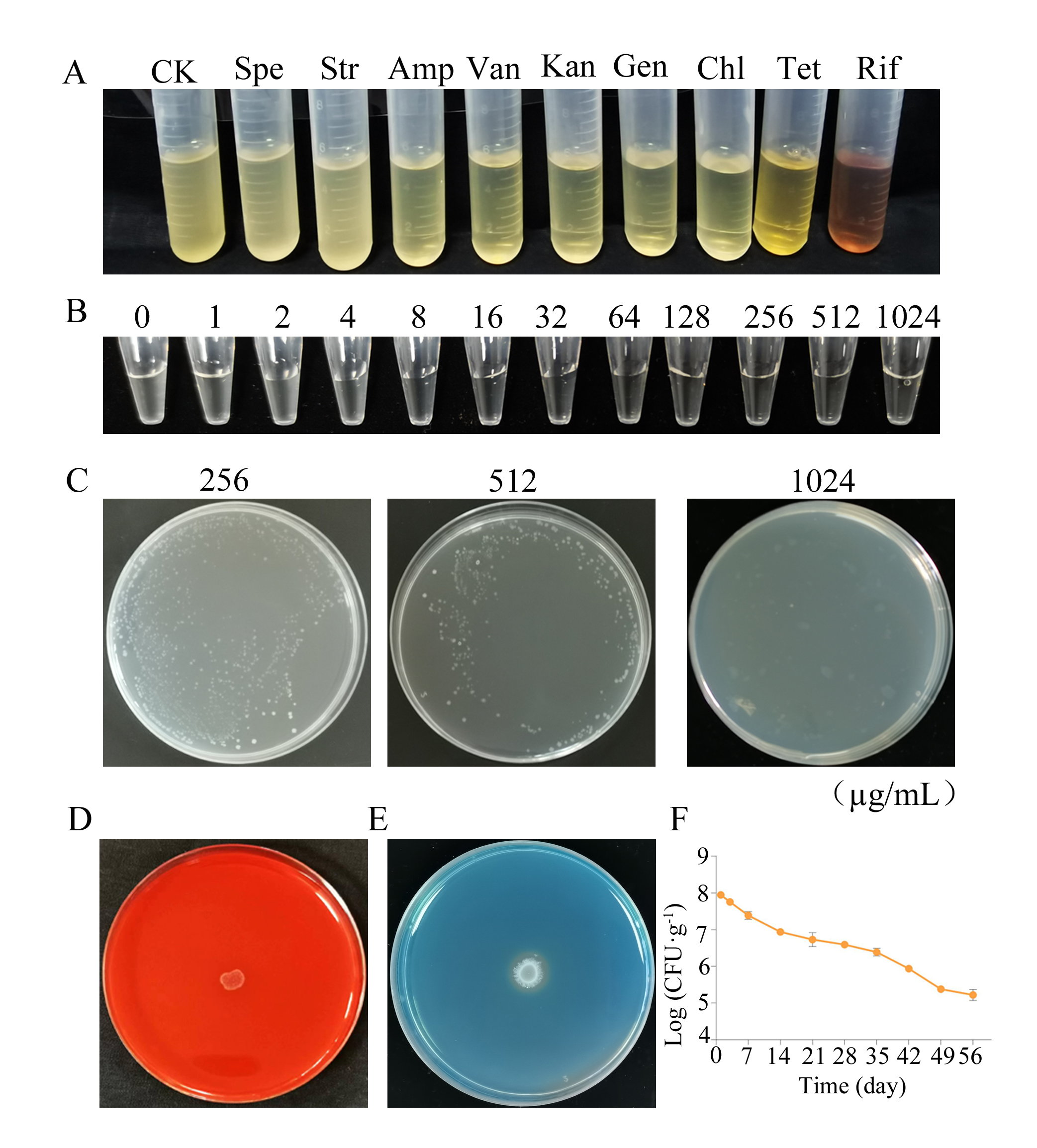

Supplement: SUPPLEMENTARY FIGURE 3 — Determination of antibiotic resistance of Paenibacillus peoriae ZBSF16. (A) Survival of P. peoriae ZBSF16 treated with different antibiotics. Spectinomycin (Spe), streptomycin (Str), ampicillin (Amp), vancomycin (Van), kanamycin (Kan), gentamycin (Gen), chloramphenicol (Chl), tetracycline (Tet) and rifampicin (Rif). (B) Minimum inhibitory concentration (MIC) of spectinomycin for strain ZBSF16. (C) Minimum bactericidal concentration (MBC) of spectinomycin for strain ZBSF16. (D) Hemolysis assay of ZBSF16. (E) Siderophores production of P. peoriae ZBSF16. (F) Population dynamics of P. peoriae ZBSF16 in the rhizosphere soil of grape. [file Image_3.jpeg]

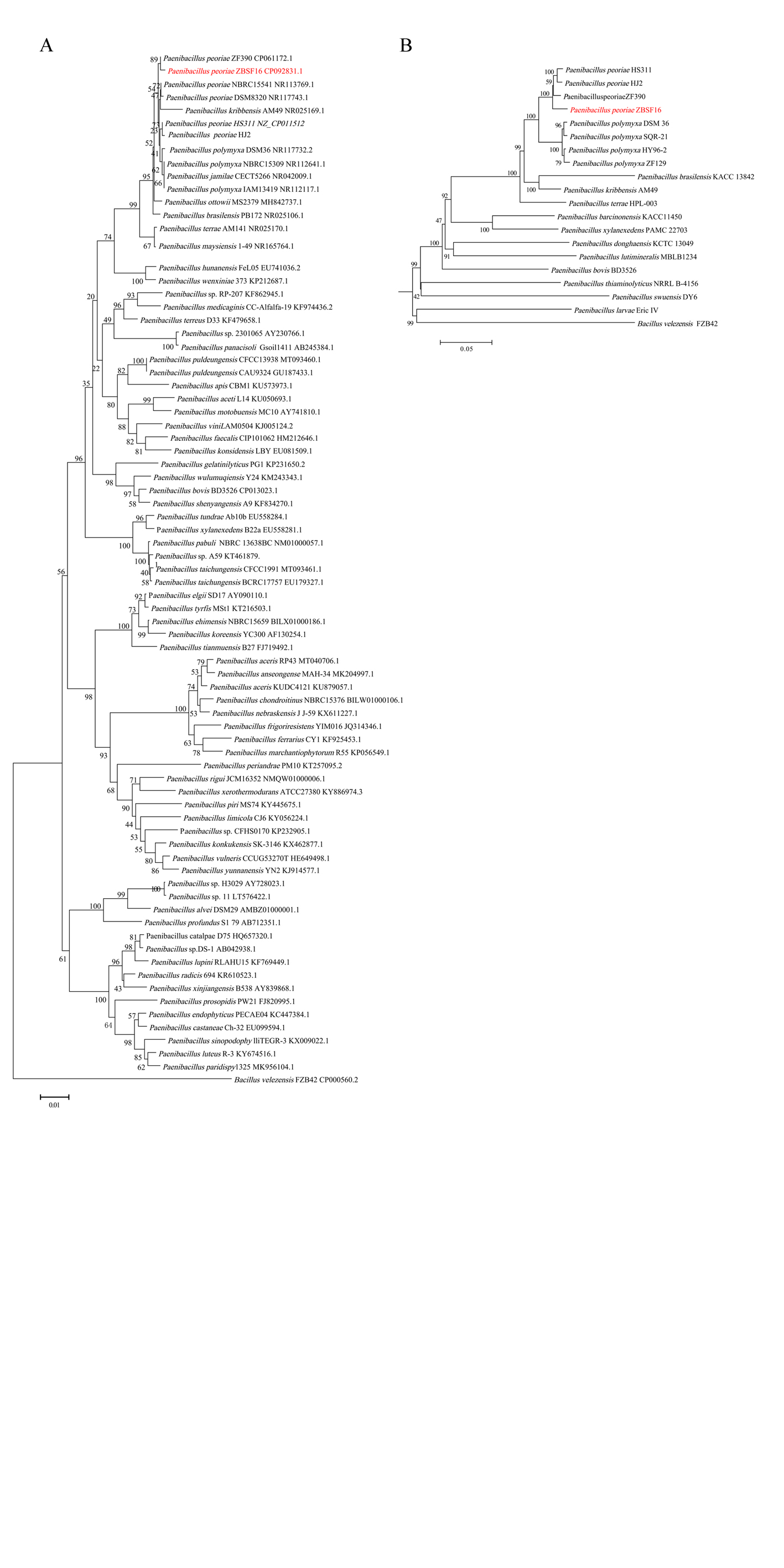

Supplement: SUPPLEMENTARY FIGURE 4 — (A) Phylogenetic tree for P. peoriae ZBSF16 and the genus Paenibacillus based on 16S rRNA (Bacillus velezensis FZB42 was used as an outgroup). (B) Phylogenetic tree of Paenibacillus peoriae ZBSF16 among other Paenibacillus species. The phylogenetic tree was constructed based on five housekeeping genes (16S rRNA, gyrB, rpoD, rho, and pgk) according to the aligned gene sequences using the maximum likelihood method in MEGA 6.0. Bootstrap values (1,000 replicates) are shown at the branch points. The scale bar indicates 0.05 nucleotide substitutions per nucleotide position. GenBank accession numbers associated with the housekeeping loci of all strains can be found in Supplementary Table 1. [file Image_4.jpeg]

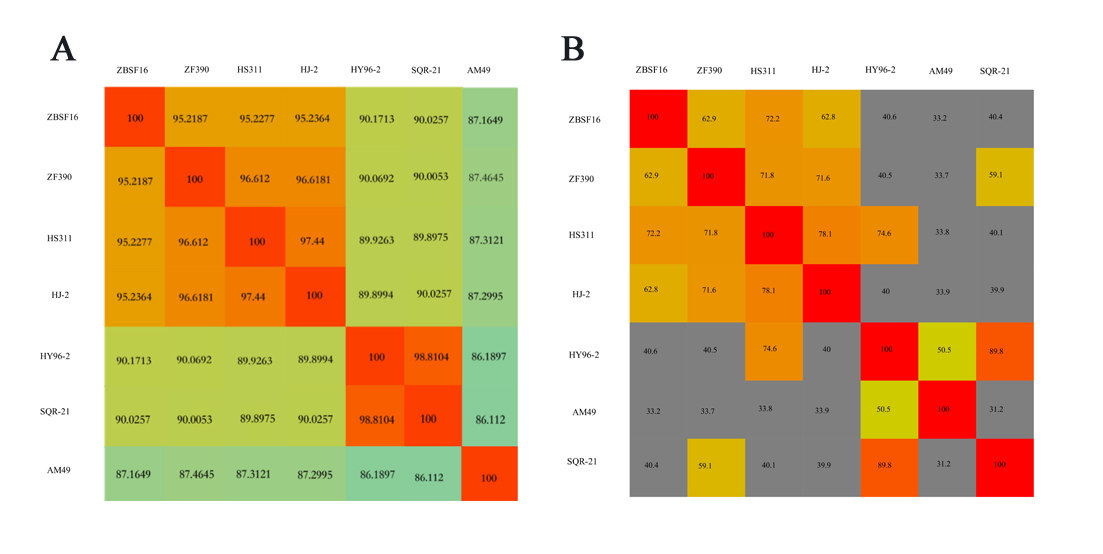

Supplement: SUPPLEMENTARY FIGURE 5 — ANI (A) and DDH (B) value matrix heatmap between Paenibacillus peoriae ZBSF16 and six other Paenibacillus genome sequences. [file Image_5.jpeg]

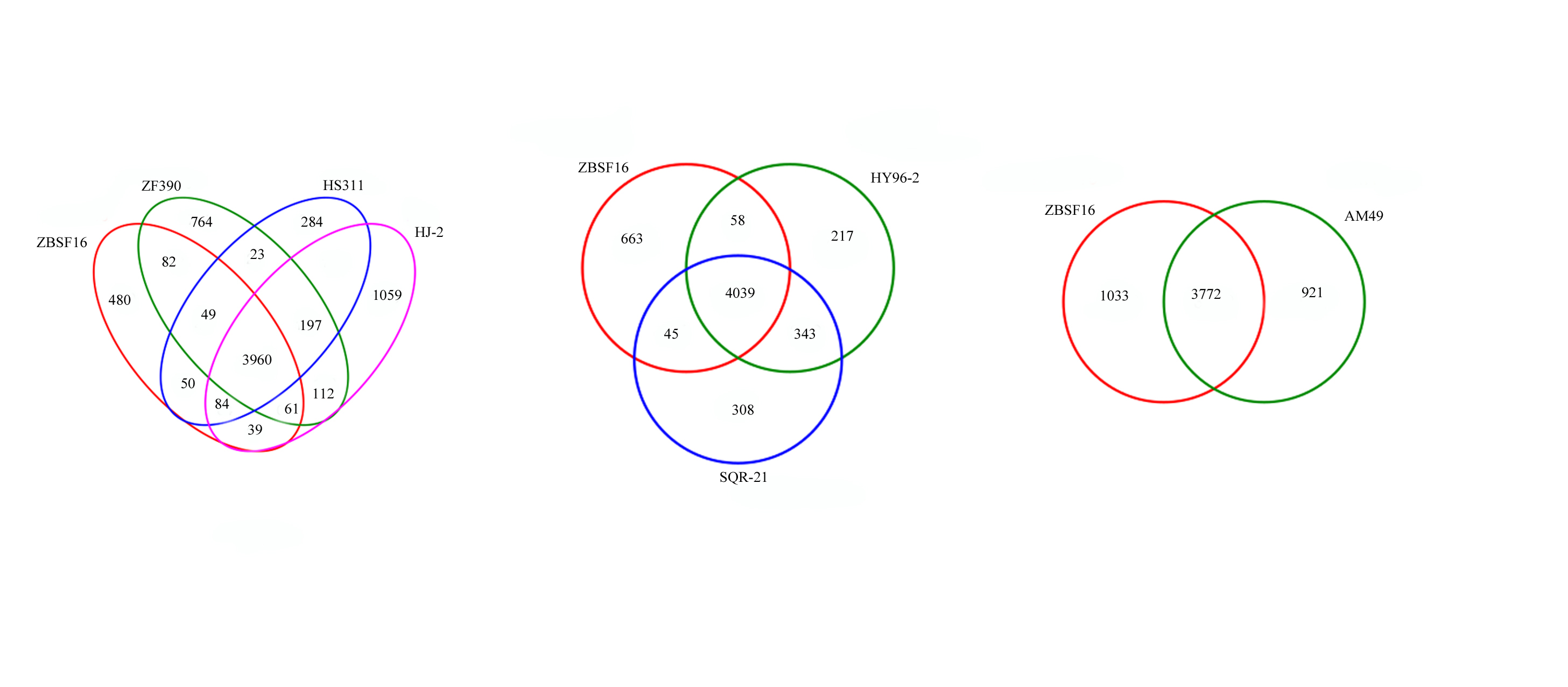

Supplement: SUPPLEMENTARY FIGURE 6 — Venn diagram showing the number of clusters of orthologous genes shared and unique genes. [file Image_6.jpeg]
