# Supplementary material for: Comparative genomic and functional analyses of Paenibacillus peoriae ZBSF16 with biocontrol potential against grapevine diseases, provide insights into its genes related to plant growth-promoting and biocontrol mechanisms
Source: Front Microbiol. 2022 Sep 8;13:975344. doi: 10.3389/fmicb.2022.975344 (PMC9492885; doi:10.3389/fmicb.2022.975344)
Supplement: Supplementary file 7 [file Table_1.DOC]

**Supplementary Table 1 Information of strains and six housekeeping genes used for phylogenetic tree construction in this study.**

| **Strain** | **16S rRNA**  **16S ribosomal RNA** | ***gyrB***  **DNA gyrase subunit beta** | ***atpD***  **ATP synthase subunit beta** | ***rpoD***  **RNA polymerase sigma factor, sigma 70** | ***rho***  ***transcription termination factor*** | ***pgk***  **Phosphoglycerate kinase** |
| --- | --- | --- | --- | --- | --- | --- |
| *Paenibacillus peoriae* ZBSF16 | MLD56_00420 | MLD56_00030 | MLD56_23370 | MLD56_16845 | MLD56_00700 | MLD56_00955 |
| *Paenibacillus peoriae* ZF390 | IAQ67_00050 | IAQ67_00030 | IAQ67_25910 | IAQ67_19485 | IAQ67_00730 | IAQ67_00990 |
| *Paenibacillus peoriae* HS311 | ABE82_00055 | ABE82_14515 | ABE82_23395 | ABE82_16910 | ABE82_00715 | ABE82_00965 |
| *Paenibacillus peoriae* HJ-2 | / | / | / | / | / | / |
| *Paenibacillus brasilensis* KACC 13842 | GCU48_RS04345 | GCU48_RS10820 | GCU48_RS08230 | GCU48_RS01780 | GCU48_RS11510 | GCU48_RS11765 |
| *Paenibacillus kribbensis* AM49 | B4V02_RS02675 | B4V02_RS25190 | B4V02_RS02005 | B4V02_RS08515 | B4V02_RS24540 | B4V02_24300 |
| *Paenibacillus polymyxa* DSM 36 | G7035_04510 | G7035_08355 | G7035_10705 | G7035_17095 | G7035_07695 | G7035_07440 |
| *Paenibacillus polymyxa* HY96-2 | C1A50_0012 | C1A50_0007 | C1A50_4826 | C1A50_3447 | C1A50_0147 | C1A50_0197 |
| *Paenibacillus polymyxa* SQR-21 | PPSQR21_008120 | PPSQR21_000060 | PPSQR21_047030 | PPSQR21_033900 | PPSQR21_001380 | PPSQR21_001890 |
| *Paenibacillus larvae* subsp. *larvae* Eric_IV | ERICIV_00009 | ERICIV_00006 | ERICIV_00053 | ERICIV_02336 | ERICIV_04306 | ERICIV_00299 |
| *Paenibacillus thiaminolyticus* NRRL B-4156 | FLT43_13705 | FLT43_16950 | FLT43_14905 | FLT43_25945 | FLT43_17700 | FLT43_18110 |
| *Paenibacillus polymyxa* ZF129 | FGY93_04045 | FGY93_08205 | FGY93_10565 | FGY93_16990 | FGY93_07510 | FGY93_07255 |
| *Paenibacillus donghaensis* KCTC 13049 | B9T62_35485 | B9T62_32155 | B9T62_29515 | B9T62_24040 | B9T62_32695 | B9T62_33015 |
| *Paenibacillus terrae* HPL-003 | HPL003_r28210 | HPL003_07520 | HPL003_04750 | HPL003_25400 | HPL003_08170 | HPL003_08385 |
| *Paenibacillus barcinonensis* KACC11450 | HUB98_10805 | HUB98_17115 | HUB98_14370 | HUB98_08380 | HUB98_17690 | HUB98_17980 |
| *Paenibacillus xylanexedens* PAMC 22703 | BS614_05080 | BS614_03045 | BS614_00200 | BS614_24690 | BS614_03645 | BS614_03940 |
| *Paenibacillus bovis* BD3526 | AR543_19870 | AR543_02900 | AR543_22650 | AR543_18055 | AR543_03365 | AR543_03655 |
| *Paenibacillus lutimineralis* MBLB1234 | EI981_00050 | EI981_00030 | EI981_26585 | EI981_08400 | EI981_00595 | EI981_01055 |
| *Paenibacillus swuensis* strain DY6 | SY83_17340 | SY83_18220 | SY83_10735 | SY83_14825 | SY83_05865 | SY83_11870 |
| *Bacillus amyloliquefaciens* FZB42 | RBAM_000080 | RBAM_000060 | RBAM_033970 | RBAM_023510 | RBAM_034240 | RBAM_031290 |
