# Supplementary material for: Comparative genomic and functional analyses of Paenibacillus peoriae ZBSF16 with biocontrol potential against grapevine diseases, provide insights into its genes related to plant growth-promoting and biocontrol mechanisms
Source: Front Microbiol. 2022 Sep 8;13:975344. doi: 10.3389/fmicb.2022.975344 (PMC9492885; doi:10.3389/fmicb.2022.975344)
Supplement: Supplementary file 8 [file Table_2.DOC]

**Supplementary Table 2 Biolog assays of** ***Paenibacillus peoriae* ZBSF16**

| No. | Test items | Result | No. | Test items | Result |
| --- | --- | --- | --- | --- | --- |
| 1 | Ala-Phe-Pro-arylamidase | - | 25 | Saccharose/Sucrose | + |
| 2 | Adonitol | - | 26 | D-Tagatose | - |
| 3 | L-Pyrrolydonyl-Arylamidase | - | 27 | D-Trehalose | + |
| 4 | L-Arabitol | - | 28 | Citrate(Sodium) | - |
| 5 | D-Cellobiose | + | 29 | Malonate | - |
| 6 | Beta-Galactosidase | + | 30 | 5-Keto-D-Gluconate | - |
| 7 | H_2_S Production | - | 31 | L-Lactate alkalinisation | - |
| 8 | Beta-N-Acetyl-Glucosaminidase | - | 32 | Alpha-Glucosidase | - |
| 9 | Glutamyl Arylamidase pNA | - | 33 | Succinate alkalinisation | - |
| 10 | D-Glucose | + | 34 | Beta-N-Acetyl-Galactosaminidase | - |
| 11 | Gamma-Glutamyl-Transferase | - | 35 | Alpha-Galactosidase | + |
| 12 | Fermentation/ Glucose | - | 36 | Phosphatase | - |
| 13 | Beta-Glucosidase | + | 37 | Glycine Arylamidase | - |
| 14 | D-Maltose | + | 38 | Ornithine Decarboxylase | - |
| 15 | D-Mannitol | + | 39 | Lysine Decarboxylase | - |
| 16 | D-Mannose | + | 40 | L-Histidine assimilation | - |
| 17 | Beta-Xylosidase | - | 41 | Courmarate | + |
| 18 | Beta-Alanine arylamidase pNA | - | 42 | Beta-Glucoronidase | - |
| 19 | L-Proline Arylamidase | - | 43 | O/129 Resistance (comp.vibrio.) | - |
| 20 | Lipase | - | 44 | Glu-Gly-Arg-Arylamidase | - |
| 21 | Palatinose | + | 45 | L-Malate assimilation | - |
| 22 | Tyrosine Arylamidase | - | 46 | Ellman | - |
| 23 | Urease | - | 47 | L-Lactate assimilation | - |
| 24 | D-Sorbitol | - |  |  |  |

Note: + and – indicate positive and negative responses, respectively.
