# Supplementary material for: Comparative genomic and functional analyses of Paenibacillus peoriae ZBSF16 with biocontrol potential against grapevine diseases, provide insights into its genes related to plant growth-promoting and biocontrol mechanisms
Source: Front Microbiol. 2022 Sep 8;13:975344. doi: 10.3389/fmicb.2022.975344 (PMC9492885; doi:10.3389/fmicb.2022.975344)
Supplement: Supplementary file 9 [file Table_3.DOC]

**Supplementary Table 3 Statistics of the genome assembly of** ***Paenibacillus peoriae* ZBSF16.**

| **Attribute** | **Value** |
| --- | --- |
| Scaffold Length (bp) | 5839239 |
| Scaffold Number | 1 |
| Scaffold N50 (bp) | 5839239 |
| Scaffold N90 (bp) | 5839239 |
| Contig Length (bp) | 5839239 |
| Contig Number | 1 |
| Contig N50 (bp) | 5839239 |
| Contig N90 (bp) | 5839239 |
| GC content (%) | 45.62 |
| Gaps Number | 0 |
