# Supplementary material for: Comparative genomic and functional analyses of Paenibacillus peoriae ZBSF16 with biocontrol potential against grapevine diseases, provide insights into its genes related to plant growth-promoting and biocontrol mechanisms
Source: Front Microbiol. 2022 Sep 8;13:975344. doi: 10.3389/fmicb.2022.975344 (PMC9492885; doi:10.3389/fmicb.2022.975344)
Supplement: Supplementary file 10 [file Table_4.DOC]

**Supplementary Table 4 Genome statistics of** ***Paenibacillus peoriae* ZBSF16.**

| **Attribute** | **Value** | **% of total** |
| --- | --- | --- |
| Genome size (bp) | 5839239 | 100.00 |
| DNA coding (bp) | 4998840 | 85.61 |
| DNA G + C (bp) | 2663860 | 45.62 |
| Genomics Islands | 164713 | 2.82 |
| Total genes | 5188 | 100.00 |
| Protein coding genes | 4,944 | 95.30 |
| RNA genes | 152 | 2.93 |
| Pseudo genes | 92 | 1.77 |
| Genes assigned to KEGGs | 2427 | 46.78 |
| Genes assigned to SwissProt | 2948 | 56.82 |
| Genes assigned to eggNOG | 4334 | 83.54 |
| Genes assigned to GOs | 3781 | 72.88 |
| Genes assigned to Pfam | 4229 | 81.52 |
| Genes assigned to NR | 5,072 | 97.76 |
| Genes assigned to TCDB | 1407 | 27.12 |
| Genes assigned to PHI | 1641 | 31.63 |
| Genes assigned to VFDB | 999 | 19.26 |
| Genes assigned to Secretory | 342 | 6.59 |
| Genes assigned to CAZY | 354 | 6.82 |
| Genes assigned to T3SS | 226 | 4.36 |
| Genes assigned to CARD | 1 | 0.02 |
| Genes with signal peptides | 415 | 8.00 |
| Genes with transmembrane helices | 1404 | 27.06 |
| Genes with Secreted Protein | 415 | 8.00 |
| GIs number | 11 | N/D |
| Prophage_Num | 10 | N/D |
| CRISPR_Num | 4 | N/D |

^a^N/D = not determined
