# Supplementary material for: Comparative genomic and functional analyses of Paenibacillus peoriae ZBSF16 with biocontrol potential against grapevine diseases, provide insights into its genes related to plant growth-promoting and biocontrol mechanisms
Source: Front Microbiol. 2022 Sep 8;13:975344. doi: 10.3389/fmicb.2022.975344 (PMC9492885; doi:10.3389/fmicb.2022.975344)
Supplement: Supplementary file 11 [file Table_5.DOC]

**Supplementary Table 5 Genes associated with flagella biosynthesis in *Paenibacillus peoriae* ZBSF16 and other *P. peoriae* strains*.***

| **Genes** | **Product Definition** | ***P. peoriae* ZBSF16** | | ***P. peoriae* ZF390** | | ***P. peoriae* HS311** | | ***P. peoriae* HJ-2** | |
| --- | --- | --- | --- | --- | --- | --- | --- | --- | --- |
|  |  | **Locus Tag** | **Protein ID** | **Protein ID** | **Homology (%)** | **Protein ID** | **Homology (%)** | **Protein ID** | **Homology (%)** |
| *flhB* | Flagellar biosynthesis protein FlhB | MLD56_09980 | UMY57307.1 | WP_007430040.1 | 97.57 | WP_007430040.1 | 97.57 | NA | 98.37 |
| *flhA* | Flagellar biosynthesis protein FlhA | MLD56_09985 | UMY56737.1 | WP_014281096.1 | 99.26 | WP_014281096.1 | 99.26 | NA | 99.85 |
| *flhF* | Flagellar biosynthesis protein FlhF | MLD56_09990 | UMY56738.1 | WP_013370692.1 | 90.44 | WP_013309905.1 | 95.83 | NA | 95.21 |
| *flgB* | Flagellar basal body rod protein FlgB | MLD56_09870 | UMY56715.1 | WP_013309883.1 | 100 | WP_013309883.1 | 100 | NA | 74.07 |
| *flgC* | Flagellar basal body rod protein FlgC | MLD56_09875 | UMY56716.1 | WP_013309884.1 | 99.33 | WP_013309884.1 | 99.33 | NA | 98.67 |
| *flgF* | Flagellar basal-body rod protein FlgF | MLD56_09885 | UMY56718.1 | WP_007430021.1 | 96.39 | WP_014281076.1 | 96.78 | NA | 93.55 |
| *flgH* | Flagellar basal body L-ring protein | MLD56_09895 | UMY56720.1 | WP_014281078.1 | 94.24 | WP_014281078.1 | 94.24 | NA | 93.55 |
| *flgI* | Flagellar basal body P-ring protein | MLD56_09900 | UMY56721.1 | WP_014281079.1 | 99.09 | WP_014281079.1 | 99.09 | NA | 99.54 |
| *flgJ* | Flagellar rod assembly protein/muramidase FlgJ | MLD56_09905 | UMY56722.1 | WP_013309890.1 | 99.32 | WP_013309890.1 | 99.32 | NA | 99.32 |
| *flgK* | Flagellar hook-associated protein FlgK | MLD56_09915 | UMY56724.1 | WP_017427926.1 | 91.33 | WP_013309892.1 | 96.30 | NA | 97.74 |
| *flgL* | Flagellar hook-associated protein FlgL | MLD56_23175 | UMY54392.1 | WP_016822919.1 | 96.73 | NA | NA | NA | NA |
| *fliA* | Flagellar biosynthesis sigma factor | MLD56_10030 | UMY56745.1 | WP_017689160.1 | 88.55 | WP_018883381.1 | 91.22 | NA | 92.37 |
| *fliD* | Flagellar capping protein | MLD56_23120 | UMY54381.1 | WP_013373375.1 | 58.00 | NA | NA | NA | NA |
| *fliS* | flagellar export chaperone FliS | MLD56_23115 | UMY54380.1 | WP_013373374.1 | 73.60 | NA | NA | NA | NA |
| *fliE* | Flagellar hook-basal body protein FliE | MLD56_09880 | UMY56717.1 | WP_014281075.1 | 97.12 | WP_014281075.1 | 97.12 | NA | 99.04 |
| *fliF* | Flagellar M-ring protein FliF | MLD56_09885 | UMY56718.1 | WP_007430021.1 | 96.39 | WP_014281076.1 | 96.78 | NA | 93.55 |
| *fliG* | Flagellar motor switch protein FliG | MLD56_09890 | UMY56719.1 | WP_015736330.1 | 92.31 | WP_015736330.1 | 92.31 | NA | 94.41 |
| *fliH* | flagellar assembly protein FliH | MLD56_09895 | UMY56720.1 | WP_014281078.1 | 94.24 | WP_014281078.1 | 94.24 | NA | 98.56 |
| *fliI* | flagellar protein export ATPase FliI | MLD56_09900 | UMY56721.1 | WP_014281079.1 | 99.09 | WP_014281079.1 | 99.09 | NA | 99.54 |
| *fliJ* | flagellar export protein FliJ | MLD56_09905 | UMY56722.1 | WP_013309890.1 | 99.32 | WP_013309890.1 | 99.32 | NA | 99.32 |
| *fliK* | Flagellar hook-length control protein-like protein | MLD56_09915 | UMY56724.1 | WP_017427926.1 | 88.67 | WP_013309892.1 | 96.44 | NA | 97.74 |
| *fliL* | flagellar basal body-associated FliL family protein | MLD56_09940 | UMY56729.1 | WP_013309897.1 | 99.36 | WP_013309897.1 | 99.36 | NA | 100 |
| *fliM* | Flagellar motor switch protein FliM | MLD56_09945 | UMY56730.1 | WP_018883398.1 | 89.46 | WP_014599663.1 | 99.70 | NA | 99.40 |
| *fliO* | Flagellar biosynthetic protein FliO | MLD56_09960 | UMY56733.1 | WP_016820958.1 | 97.75 | WP_007430036.1 | 96.61 | NA | 99.44 |
| *fliP* | flagellar type III secretion system pore protein FliP | MLD56_09965 | UMY56734.1 | WP_013309901.1 | 100 | WP_013309901.1 | 100 | NA | 99.60 |
| *fliQ* | Flagellar biosynthetic protein FliQ | MLD56_09970 | UMY56735.1 | WP_010346369.1 | 98.88 | WP_010346369.1 | 98.88 | NA | 100 |
| *fliR* | flagellar type III secretion system protein FliR" | MLD56_09975 | UMY56736.1 | WP_013309902.1 | 99.62 | WP_013309902.1 | 99.62 | NA | 100 |
| *FliY* | flagellar motor switch phosphatase FliY | MLD56_09950 | UMY56731.1 | WP_016820957.1 | 98.70 | WP_013309899.1 | 99.57 | NA | 96.86 |
| *MotB* | flagellar motor protein MotB | MLD56_00505 | UMY55015.1 | WP_016818543.1 | 95.27 | WP_013308184.1 | 99.64 | NA | 93.99 |
| *MotA* | flagellar motor stator protein MotA | MLD56_00510 | UMY55016.1 | WP_007428081.1 | 95.87 | WP_014279092.1 | 98.76 | NA | 100 |
| */* | flagellar hook capping protein | MLD56_09920 | UMY56725.1 | WP_017427927.1 | 96.55 | WP_013309893.1 | 98.63 | NA | 92.66 |
| */* | flagellar biosynthesis protein | MLD56_09925 | UMY56726.1 | WP_019686990.1 | 94.44 | WP_019686990.1 | 94.44 | NA | 97.62 |

NA = not available.

/ = not determined.
