# Supplementary material for: Comparative genomic and functional analyses of Paenibacillus peoriae ZBSF16 with biocontrol potential against grapevine diseases, provide insights into its genes related to plant growth-promoting and biocontrol mechanisms
Source: Front Microbiol. 2022 Sep 8;13:975344. doi: 10.3389/fmicb.2022.975344 (PMC9492885; doi:10.3389/fmicb.2022.975344)
Supplement: Supplementary file 12 [file Table_6.DOC]

**Supplementary Table 6 Genes related to biofilm in** ***Paenibacillus peoriae* ZBSF16 other *P. peoriae* strains.**

| **Genes** | **Product Definition** | ***P. peoriae* ZBSF16** | | ***P. peoriae* ZF390** | | ***P. peoriae* HS311** | | ***P. peoriae* HJ-2** | |
| --- | --- | --- | --- | --- | --- | --- | --- | --- | --- |
|  |  | **Locus Tag** | **Protein ID** | **Protein ID** | **Homology (%)** | **Protein ID** | **Homology (%)** | **Protein ID** | **Homology (%)** |
| *spo0A_2* | stage 0 sporulation family protein | MLD56_00105 | UMY54943.1 | WP_014279023.1 | 99.25 | WP_014279023.1 | 99.25 | N/A | 98.50 |
| *yabG* | sporulation peptidase YabG | MLD56_00155 | UMY57231.1 | WP_013308119.1 | 98.99 | WP_013308119.1 | 98.99 | N/A | 100 |
| *spoVT* | stage V sporulation protein T | MLD56_00215 | UMY54964.1 | WP_014279043.1 | 98.33 | WP_014279043.1 | 98.33 | N/A | 99.44 |
| *yabP* | sporulation protein YabP | MLD56_00240 | UMY54969.1 | WP_007428030.1 | 95.74 | WP_007428030.1 | 95.74 | N/A | 100 |
| *spoIIE* | stage II sporulation protein E | MLD56_00260 | UMY54973.1 | WP_019685739.1 | 97.72 | WP_019685739.1 | 97.72 | N/A | 99.75 |
| *ypdA_1* | sensor histidine kinase | MLD56_01140 | UMY55120.1 | WP_017425626.1 | 97.35 | WP_017425626.1 | 97.35 | N/A | 98.68 |
| *yehU_1* | sensor histidine kinase | MLD56_01860 | UMY55227.1 | WP_013308420.1 | 97.16 | WP_013308420.1 | 97.16 | N/A | 96.48 |
| *ypdA_2* | sensor histidine kinase | MLD56_02890 | UMY55421.1 | WP_007428560.1 | 90.23 | WP_007428560.1 | 90.23 | N/A | 97.35 |
| *glnK* | sensor histidine kinase | MLD56_01240 | UMY55139.1 | WP_013368943.1 | 88.97 | WP_013368943.1 | 88.97 | N/A | 97.47 |
| *spo0F* | response regulator | MLD56_00685 | UMY57239.1 | WP_013308218.1 | 100 | WP_013308218.1 | 100 | N/A | 100 |
| *spo0A_1* | response regulator | MLD56_01235 | UMY55138.1 | WP_013308322.1 | 100 | WP_013308322.1 | 100 | N/A | 99.68 |
| *abrB* | transcriptional regulator | MLD56_00125 | UMY54947.1 | WP_019006085.1 | 92.86 | WP_019006085.1 | 92.86 | N/A | 100 |

NA = not available.
