# Supplementary material for: Comparative genomic and functional analyses of Paenibacillus peoriae ZBSF16 with biocontrol potential against grapevine diseases, provide insights into its genes related to plant growth-promoting and biocontrol mechanisms
Source: Front Microbiol. 2022 Sep 8;13:975344. doi: 10.3389/fmicb.2022.975344 (PMC9492885; doi:10.3389/fmicb.2022.975344)
Supplement: Supplementary file 13 [file Table_7.DOC]

**Supplementary Table 7 Genes related to quorum sensing in** ***Paenibacillus peoriae* ZBSF16 and other *P. peoriae* strains.**

| **Genes** | **Product Definition** | ***P. peoriae* ZBSF16** | | ***P. peoriae* ZF390** | | ***P. peoriae* HS311** | | ***P. peoriae* HJ-2** | |
| --- | --- | --- | --- | --- | --- | --- | --- | --- | --- |
|  |  | **Locus Tag** | **Protein ID** | **Protein ID** | **Homology (%)** | **Protein ID** | **Homology (%)** | **Protein ID** | **Homology (%)** |
| */* | LysR family transcriptional regulator | NA | NA | WP_014278051.1 | NA | NA | NA | NA | NA |
| *luxS* | S-ribosylhomocysteine lyase | MLD56_03000 | UMY55442.1 | WP_007428584.1 | 99.35 | WP_007428584.1 | 99.35 | NA | 99.35 |
| *ydiK* | AI-2E family transporter | MLD56_03525 | UMY55541.1 | WP_013369319.1 | 94.63 | WP_013369319.1 | 94.63 | NA | 88.49 |
| *tqsA* | AI-2E family transporter | MLD56_06325 | UMY56057.1 | WP_017426473.1 | 98.06 | WP_017426473.1 | 98.06 | NA | 99.45 |
| *perM* | AI-2E family transporter | MLD56_19050 | UMY53652.1 | WP_013372599.1 | 97.92 | WP_013311516.1 | 99.22 | NA | 99.22 |
| */* | AI-2E family transporter | MLD56_19105 | UMY53662.1 | WP_019688314.1 | 96.34 | WP_013311527.1 | 97.46 | NA | 98.31 |
| */* | I-2E family transporter | MLD56_23635 | UMY54482.1 | WP_010347216.1 | 91.99 | WP_013312375.1 | 97.42 | NA | 97.93 |

NA = not available.

/= indefinite.
