# Supplementary material for: Comparative genomic and functional analyses of Paenibacillus peoriae ZBSF16 with biocontrol potential against grapevine diseases, provide insights into its genes related to plant growth-promoting and biocontrol mechanisms
Source: Front Microbiol. 2022 Sep 8;13:975344. doi: 10.3389/fmicb.2022.975344 (PMC9492885; doi:10.3389/fmicb.2022.975344)
Supplement: Supplementary file 14 [file Table_8.DOC]

**Supplementary Table 8 Genes associated with the chemtaxis and two-component system in *Paenibacillus peoriae* ZBSF16 and other *P. peoriae* strains.**

| **Genes** | **Product Definition** | ***P. peoriae* ZBSF16** | | ***P. peoriae* ZF390** | | ***P. peoriae* HS311** | | ***P. peoriae* HJ-2** | |
| --- | --- | --- | --- | --- | --- | --- | --- | --- | --- |
|  |  | **Locus Tag** | **Protein ID** | **Protein ID** | **Homology (%)** | **Protein ID** | **Homology (%)** | **Protein ID** | **Homology (%)** |
| *CheA* | sensor kinase CheA | MLD56_10005 | UMY56740.1 | WP_016820967.1 | 97.42 | WP_013309908.1 | 97.56 | NA | 98.57 |
| *CheW* | chemotaxis protein CheW | MLD56_10010 | UMY56741.1 | WP_013370696.1 | 99.35 | WP_013370696.1 | 99.35 | NA | 100.00 |
| *CheC* | chemotaxis protein CheC | MLD56_10015 | UMY56742.1 | WP_010346360.1 | 97.12 | WP_010346360.1 | 97.12 | NA | 98.56 |
| *CheD* | chemotaxis protein CheD | MLD56_10020 | UMY56743.1 | WP_007430048.1 | 98.79 | WP_007430048.1 | 98.79 | NA | 100.00 |
| *CheR* | protein-glutamate O-methyltransferase CheR | MLD56_14635 | UMY57333.1 | WP_017687705.1 | 78.91 | WP_013310721.1 | 98.87 | NA | 96.69 |
| *CheX* | chemotaxis protein CheX | MLD56_20500 | UMY53912.1 | WP_010347881.1 | 98.03 | WP_010347881.1 | 98.03 | NA | 96.75 |
| *CitG* | triphosphoribosyl-dephospho-CoA synthase CitG | NA | NA | WP_007431742.1 | NA | WP_007431742.1 | NA | NA | NA |
| *DcuS* | sensor histidine kinase DcuS | MLD56_17045 | UMY53277.1 | WP_013311168.1 | 97.64 | NA | NA | NA | 89.51 |
| *DcuR* | response regulator DcuR | MLD56_17040 | UMY53276.1 | WP_016821613.1 | 94.47 | WP_013311167.1 | 98.30 | NA | 98.30 |
| *YycI* | two-component system regulatory protein YycI | MLD56_25705 | UMY54880.1 | WP_013312780.1 | 98.43 | WP_013312780.1 | 98.43 | NA | 97.64 |
| *YycH* | two-component system activity regulator YycH | MLD56_25710 | UMY54881.1 | WP_013312781.1 | 97.53 | WP_013312781.1 | 97.53 | NA | 96.40 |

NA = not available.
