# Supplementary material for: Comparative genomic and functional analyses of Paenibacillus peoriae ZBSF16 with biocontrol potential against grapevine diseases, provide insights into its genes related to plant growth-promoting and biocontrol mechanisms
Source: Front Microbiol. 2022 Sep 8;13:975344. doi: 10.3389/fmicb.2022.975344 (PMC9492885; doi:10.3389/fmicb.2022.975344)
Supplement: Supplementary file 15 [file Table_9.DOC]

**Supplementary Table** **9 Secondary metabolite cluster identified in *Paenibacillus peoriae* ZBSF16 *and other Paenibacillus* spp.**

| Cluster | Type | Most similar | *P. peoriae* ZBSF16 | *P. peoriae* ZF390 | *P. peoriae* HS311 | *P. peoriae* HJ-2 | *P. polymyxa* HY96-2 | *P. polymyxa* SQR-21 | *P. kribbensis* AM49 |
| --- | --- | --- | --- | --- | --- | --- | --- | --- | --- |
| Cluster 1 | NRPS | fusaricidinB | 100% | 100% | 100% | 100% | 87% | 100% | 100% |
| Cluster 2 | ranthipeptide | / | - | - | - | - | - | + | - |
| Cluster 3 | Lanthipeptide-class-i,cyclic-lactone-autoinducer | paenibacillin | 90% | - | - | - | - | - | - |
| Cluster 4 | proteusin | / | + | - | + | + | + | + | + |
| Cluster 5 | lassopeptide | paeninodin | 40% | 40% | 40% | - | + | + | 40% |
| Cluster 6 | NRPS | bacillibactin | 53% | - | - | - | - | - | 53% |
| Cluster 7 | NRPS-like | / |  | + | + | + | + | + | + |
| Cluster 8 | Cyclic-lactone-autoinducer | / | + | + | + | + | + | + | + |
| Cluster 9 | NRPS | tridecaptin | 100% | 100% | 100% | 100% | 100% | 100% | 100% |
| Cluster 10 | NRPS | paenilipoheptin | 11% | - | 11% | - | - | - | - |
| Cluster 11 | T1PKS, NRPS | brevicidine | 18% | 18% | - | - | 18% | - | - |
| Cluster 12 | NRPS, betalactone | paenilipoheptin | 7% | 69% | - | 76% | - | - | 19% |
| Cluster 13 | Cyclic-lactone-autoinducer | + | - | - | - | - | - | - | - |
| Cluster 14 | transAT-PKS, NRPS | lacunalides | 19% | - | + | - | + | + | - |
| Cluster 15 | NRPS | polymyxin | 100% | 100% | 100% | - | 100% | 100% | - |
| Cluster 16 | lanthipeptide-class-i | Paenicidin B | 100% | - | 57% | - | - | 100% | - |
| Cluster 17 | lassopeptide, RRE-containing | / | + | - | + | + | - | - | - |
|  | lanthipeptide-class-i | paenilan | - | 100% | - | 100% | 90% | 100% | - |
|  | siderophore | / | - | + | + | + | - | - | - |
|  |  | pelgipeptin | - | - | 25% | 50% | - | - | - |
|  | transAT-PKS, NRPS, T3PKS, PKS-like | aurantininB/C/D | - | - | 35% | - | 32% | - | - |
|  | phosphonate | / | - | - | + | + | - | - | - |
|  |  | bacillaene | - | - | - | 85% | - | - | - |
|  | phosphonate | tauramamide | - | - | - | 9% | - | - | 9% |
|  |  | marthiapeptideA | - | - | - | - | 41% | 41% | - |
|  |  | brevicidine | - | - | - | - | 18% | 18% | - |
|  | betalactone | Anabaenopeptin NZ857/nostamideA | - | - | - | - | 100% | 100% | - |
|  | Lanthipeptide-class-i | S-layer glycan | - | - | - | - | 33% | 28% | - |
|  | Lanthipeptide-class-i | Paenicidin A | - | - | - | - | 85% | - | - |
|  |  | bacillomycinFD | - | - | - | - | - | + | - |
|  | transAT-PKS, T3PKS, PKS-like, NRPS | kalimantacinA | - | - | - | - | - | 27% | - |
|  |  | macrobrevin | - | - | - | - | - | - | 20% |
|  |  | ataphylobactin | - | - | - | - | - | - | + |
|  | NRPS | Bacillomycin D | - | - | - | - | - | 20% | - |
|  | terpene | / | - | - | - | - | - | - | + |

“/”represents not determined; “+” represents the cluster can be found in the strain; “-” represents the cluster is absent in the strain.

+= positive
